# Proteomic characterisation of perhexiline treatment on THP-1 M1 macrophage differentiation

**DOI:** 10.3389/fimmu.2023.1054588

**Published:** 2023-03-13

**Authors:** Bimala Dhakal, Celine Man Ying Li, Mahnaz Ramezanpour, Ghais Houtak, Runhao Li, George Bouras, Alex Collela, Nusha Chegeni, Tim Kennion Chataway, Paul Drew, Benedetta C. Sallustio, Sarah Vreugde, Eric Smith, Guy Maddern, Giovanni Licari, Kevin Fenix

**Affiliations:** ^1^ Discipline of Surgery, Adelaide Medical School, The University of Adelaide, Adelaide, SA, Australia; ^2^ The Basil Hetzel Institute for Translational Health Research, The Queen Elizabeth Hospital, Adelaide, SA, Australia; ^3^ Department of Surgery-Otolaryngology Head and Neck Surgery, Central Adelaide Local Health Network, Adelaide, SA, Australia; ^4^ Medical Oncology, The Queen Elizabeth Hospital, Adelaide, SA, Australia; ^5^ Flinders Omics Facility, Department of Human Physiology, Flinders University, Adelaide, SA, Australia; ^6^ Discipline of Pharmacology, School of Biomedicine, The University of Adelaide, Adelaide, SA, Australia

**Keywords:** M1 macrolphage, perhexiline, quantitative proteomics, THP-1 derived macrophages, macrophage polarisation

## Abstract

**Background:**

Dysregulated inflammation is important in the pathogenesis of many diseases including cancer, allergy, and autoimmunity. Macrophage activation and polarisation are commonly involved in the initiation, maintenance and resolution of inflammation. Perhexiline (PHX), an antianginal drug, has been suggested to modulate macrophage function, but the molecular effects of PHX on macrophages are unknown. In this study we investigated the effect of PHX treatment on macrophage activation and polarization and reveal the underlying proteomic changes induced.

**Methods:**

We used an established protocol to differentiate human THP-1 monocytes into M1 or M2 macrophages involving three distinct, sequential stages (priming, rest, and differentiation). We examined the effect of PHX treatment at each stage on the polarization into either M1 or M2 macrophages using flow cytometry, quantitative polymerase chain reaction (qPCR) and enzyme linked immunosorbent assay (ELISA). Quantitative changes in the proteome were investigated using data independent acquisition mass spectrometry (DIA MS).

**Results:**

PHX treatment promoted M1 macrophage polarization, including increased *STAT1* and *CCL2* expression and IL-1β secretion. This effect occurred when PHX was added at the differentiation stage of the M1 cultures. Proteomic profiling of PHX treated M1 cultures identified changes in metabolic (fatty acid metabolism, cholesterol homeostasis and oxidative phosphorylation) and immune signalling (Receptor Tyrosine Kinase, Rho GTPase and interferon) pathways.

**Conclusion:**

This is the first study to report on the action of PHX on THP-1 macrophage polarization and the associated changes in the proteome of these cells.

## Introduction

Macrophages are heterogenous innate immune cells critical in shaping the local immune microenvironment. Activated macrophages have been subdivided into two cell states based on the cytokine and other factors to which they have been exposed during differentiation. Classically activated M1 macrophages are polarized by microbial products including lipopolysaccharides (LPS) or T helper (Th) 1 released cytokines such as interferon gamma (IFN-γ). M1 macrophages eliminate microbial pathogens by phagocytosis and release type 1 inflammatory cytokines that help activate T cell responses. Alternatively activated M2 macrophages are polarised by Th-2 cytokines like interleukin 4 (IL-4) and are involved in releasing anti-inflammatory cytokines (TGF-β and IL-10), efferocytosis of dead cells, and promoting tissue regeneration (1, 2). Recent advances in single cell RNA sequencing have shown that macrophage polarization is more diverse and plastic than previously appreciated (3, 4), however classifying macrophages as proinflammatory M1 or anti-inflammatory M2 is still useful, particularly when considering the development of drugs to modulate macrophage states in disease.

Macrophage dysregulation is common in many immunopathologies (5). Autoimmune diseases are commonly associated with an increase in M1-like macrophages leading to the chronic inflammation (6). In cancer, infiltration of M2-like macrophages leads to increased anti-tumour immune suppression, angiogenesis, cancer outgrowth and metastasis (7, 8). Severe forms of chronic airway diseases such as asthma and chronic rhinosinusitis are linked with disrupted airway microbiomes (9–11) that correlate with a M2-like macrophage profile with disrupted phagocytic capacity leading to chronic inflammation at these sites (12–16).

Therapeutic interventions that target macrophages, including depletion, repolarization or functional inhibition, have been investigated in many autoimmune, chronic inflammatory diseases, and in cancers (8, 17, 18). Different macrophage subsets have characteristic metabolic pathway profiles, with M1-like macrophages increase reliance on glycolysis, M2-like macrophages fatty acid oxidation (FAO) and oxidative phosphorylation (19, 20). Drugs which target metabolic pathways to inhibit particular subsets are currently being explored as novel therapeutics for macrophage related diseases (20).

Perhexiline, (2-(2,2-dicyclohexylethyl)piperidine, PHX), is used clinically to treat ischaemic refractory angina, and its utility has also been studied in aortic stenosis and heart failure (21–23). Mechanistically, PHX has been shown to reduce FAO through the inhibition of carnitine palmitoyltransferase-1 (CPT-1), an enzyme responsible for mitochondrial uptake of long-chain fatty acids (22). It has been suggested that PHX limits M2 macrophage polarization also by inhibiting this pathway (24). Furthermore, due to its ability to activate krüppel-like factor 14 (KLF14), PHX can mitigate macrophage mediated inflammation by ultimately downregulating IL-1β (25, 26). These results suggest that PHX may be a suitable candidate drug for the management of diseases which involve dysregulation of macrophage phenotypes by ultimately shifting macrophage states. The aim of this study was to determine the effect of PHX on the differentiation of the THP-1 monocytoid cell line into M1-like and M2-like states and using DIA MS, identify the unique proteomic changes that occur in PHX treated macrophages.

## Material and methods

### Cell lines, cell culture and reagents

The THP-1 cell line was purchased from American Type Culture Collection (ATCC). The cells were cultured in complete medium consisting of RPMI 1640 Medium containing sodium bicarbonate (Sigma-Aldrich) supplemented with 10% FBS (Sigma-Aldrich), 200 U/mL penicillin, 200 µg/mL streptomycin and GlutaMAX Supplement (Life Technologies) and incubated at 37 °C with 5% CO_2_. Cells were tested for mycoplasma using the MycoAlert mycoplasma detection kit (Lonza). The THP-1 cells were differentiated into M0, M1 or M2 macrophages using a standard protocol with minor modifications (27). Briefly, 1x10^6^ THP-1 cells were seeded into 24 well plates and treated with 5 ng/mL phorbol-12-myristate-13-acetate (PMA, Sigma-Aldrich) for 24 hours. PMA was then replaced with fresh complete medium and the cells incubated for 72 hours, followed by stimulation with either 250 ng/mL lipopolysaccharide (LPS, Sigma-Aldrich) and 20 ng/mL interferon-γ (IFN-γ, Miltenyi Biotec), or 20 ng/mL interleukin-4 (IL-4, Miltenyi Biotec) for 48 hours, to differentiate them into M1 or M2 macrophages respectively. Perhexiline (PHX, Sigma-Aldrich) was added at different stages of the culture protocol i.e., during addition of PMA (priming), during resting stage for 72 hours (rest), or for 48 hours along with addition of cytokines and LPS (differentiation). For the IL-1β release experiments, cell-free supernatant was collected from M1 macrophages after differentiation. Then, the macrophages were washed twice with phosphate buffered saline (PBS). Finally, IL-1β release was further stimulated by treating with complete medium supplemented with 1 μg/mL LPS for 2 hours followed by the addition of 5 mM adenosine triphosphate (ATP, Sigma-Aldrich) for 1 hour, before the cell-free supernatant was collected. For unstimulated controls, the medium was complete medium without LPS and ATP.

### Flow cytometry

Macrophages were stained with BD Horizon Fixable Viability Stain 780 (FVS780) (Biolegend). Cells were then treated with 50 μL of FC block (BD Biosciences), then stained using anti-human CD80 A647, anti-human CD209 BV421, anti-human CD14 FITC and anti-human B2M PE conjugated antibodies (Biolegend), prepared in FACS buffer, for 30 minutes at 4 °C. After washing, the cells were resuspended in FACS buffer and analysed using a FACS Canto II flow cytometry system (BD Biosciences). The data were analysed using FlowJo v10.4.1 software (BD Biosciences).

### RNA extraction and quantitative polymerase chain reaction

Cells were washed in PBS and RNA was extracted using PureLink RNA Mini Kit (Life Technologies). RNA concentrations were measured and purity was checked using a Nanodrop-1000 spectrophotometer (Thermo Fisher Scientific). Total RNA (100 ng) was reverse transcribed using the iScript cDNA Synthesis Kit (Bio-Rad). qPCR was performed using a ViiA 7 Real-Time PCR System (Life Technologies) using TaqMan Fast Advanced Master Mix (Life Technologies). Primers (Life Technologies) were purchased for the following genes: *STAT1* (Hs01013996_m1), *STAT6* (Hs00598625_m1), *CCL2* (Hs00234140_m1), *c-MYC* (Hs00153408_m1), and *RPML37* (Hs01102345_m1) as an internal control. All qPCR analyses were performed using the ΔCT method in Design and Analysis Quant studio Software (Thermo Fisher Scientific).

### Enzyme-linked immunosorbent assay

IL-1β from cell-free supernatants were quantified using ELISA MAX™ Deluxe Set Human IL-1β kit (Biolegend), following the manufacturer’s instruction. Absorbance was measured at 450 nm using a FLUOstar Optima microplate reader (BMG Labtech).

### Cell lysate extraction for mass spectrometry analysis

Vehicle and 5 µM PHX treated M1-like macrophages were lysed in 50 mM Tris (pH 8.5) buffer containing 1X cocktail of protease inhibitors (Thermo Fisher Scientific) using manual homogenisation. Cells were centrifuged at 42,000 × g for 5 min at 4 °C. The protein content of the supernatant was estimated using a NanoOrange Protein Quantitation Kit (Thermo Fisher Scientific). Protein and peptide single-pot solid-phase-enhanced sample preparation (SP3) with Sera-Mag SpeedBead Carboxylate-Modified Magnetic Particles (Cytiva) was performed on cell lysates adopting manufacturer’s instructions. Briefly, proteins were reduced with 10 mM tris (2-carboxyethyl) phosphine (TCEP) for 30 min at 56 °C, then alkylated with 20 mM chloroacetamide in the dark for 30 min at room temperature. Hydrophobic and hydrophilic beads were resuspended 1:1 in ultra-pure water at 10 μg/μL. A 10:1 bead /protein ratio was added to lysates. Ethanol (100%) was added at 1:1 volume to the bead/protein mix and incubated for 10 min at 1000 rpm using a ThermoMixer (Eppendorf). Samples were then placed on a magnetic rack for 2 min. Supernatants were aspirated and carefully washed 3x in 80% ethanol with each wash having a 1 min magnetic separation step. Beads were then air-dried for 30 s before being resuspended in 100 mM ammonium bicarbonate. Trypsin was added in a 1:20 enzyme-to substrate ratio to each sample and incubated at 37°C overnight to digest the protein. A follow-up peptide clean-up was then performed. Following a 2 min magnetic separation step, supernatants containing peptides were transferred into a clean tube before being bound to freshly prepared Sera-Mag SpeedBeads. Acetonitrile (100%) was then immediately added to reach a final concentration ≥ 95% to initiate peptide binding to beads. Samples were then incubated for 10 min at 1000 rpm using a Thermomixer and then washed three times in acetonitrile with magnetic separation in each step. Beads were then air-dried before peptides were eluted with 2% DMSO. The concentration of the peptides was brought to 1 μg/3 μL prior to mass spectrometry acquisition. For reproducibility and to build a chromatogram library, aliquots from five replicates were pooled to ensure that the pool contained all peptides.

### Liquid chromatography

Peptides were analysed with a Dionex Ultimate 3000 UPLC coupled with a Orbitrap Exploris 480 tandem mass spectrometer (Thermo Fisher Scientific). An in-house pulled column created from 75 µm inner diameter fused silica capillary packed with 1.9 µm ReproSil-Pur C18 beads (Dr. Maisch, Ammerbuch, Germany) to 25 cm, coupled with a PepMap™ 100 trap cartridge (0.3 x 5 mm, 5 µm C18, Thermo Fisher Scientific) were used. Solvent A was 0.1% formic acid in water and solvent B was 0.1% formic acid in 80% acetonitrile. For each injection, 1 µg of peptides was loaded and separated using a 120 min gradient from 3 to 31.2% solvent B, followed by a 30 min washing and equilibration gradient.

### Spectral library generation

A pooled sample comprised of 1.5 µL of each protein digest was used to generate a sample project specific spectral library for data dependent analysis (DDA). Six gas phase fractionation (GPF) chromatogram library acquisitions, each spanning a narrow m/z range across the 350 – 1200 m/z total mass range (350-500 m/z method 1, 490-610 m/z method 2, 600-710 m/z method 3, 700-810 m/z method 4, 800-910 m/z method 5, 900-1200 m/z method 6). For each DDA-GPF analysis two µL of the pooled sample was used with a three second cycle time instrument method. Briefly, a narrow spectrum ms1 scan matching one of the six m/z mass ranges was performed using an orbitrap resolution of 60,000. A normalised AGC target of 3e6 with an auto maximum injection time mode was used. An intensity threshold of 2.5e5 and dynamic exclusion of time of 45 s was employed for all data dependent ms2 scans that were acquired at 15,000 resolution, AGC target 5e4, 33% normalised collision energy (NCE) in the HCD cell, with an auto maximum inject time mode used.

### Quantitative shotgun data independent acquisition mass spectrometry

For the Data Independent Acquisition (DIA) runs, the Orbitrap Exploris 480 was configured to acquire 37 16 m/z precursor isolation windows (396.43 –1004.70 m/z), followed by 37 16 m/z windows (400.43 –1008.70 m/z) creating a staggered window pattern. An ms2 resolution of 15,000, AGC target 5e4, maximum inject time of 20 ms, and normalised HDC collision energy of 28 was employed for all DIA scans. Precursor spectra over a 390 - 1010 m/z mass range were acquired prior to DIA scans with a resolution of 60,000, AGC target 3e5, maximum inject time 100 ms were used for all full scan mass spectrometry (MS) spectra.

### DIA data analysis

Spectronaut™ (version 15.0.210615.50606, Biognosis) was used for both spectral library generation and DIA data analysis. Factory default setting were used for all analysis steps.

### Bioinformatic analysis

Differential protein abundance analysis between the control and PHX treated groups was conducted in R using the DEqMS package (28). The number of precursors used for quantification counts and log2 abundance scores for each protein and sample were used in the analysis. A protein was determined to be present in a sample if it had at least two number of precursors, in order to guard against false positives (29). Proteins were included in the differential expression analysis if they were present in at least two samples in each group. All p-values were corrected using a false discovery rate (FDR) of 0.05.

Differentially expressed proteins were then submitted to the STRING database (30) to generate protein-protein interactions, and clusters from downregulated proteins were identified using inbuilt clustering tools with a k-means cluster of three. Gene set enrichment analysis (GSEA) of individual clusters was undertaken using the Reactome Knowledgebase (31).

Ranked GSEA was performed with the clusterProfiler package (32). Gene sets identified as significant (FDR < 0.2, p < 0.05) with GSEA were visualized using the enrichplot package (33). The following ontologies were included in the analysis of the Molecular Signatures Database (MSigDB) (34, 35): Hallmark gene set collection (36), Gene Ontology (GO) (37, 38): Biological Process, GO: Molecular Function, GO: Cellular Component, KEGG Pathway (39) and Reactome Knowledgebase (31).

### Statistical analysis

Technical and biological replicates were included in all experiments. Statistical analysis is described in figure legends.

## Results

### Perhexiline treatment promotes M1 macrophage differentiation

To generate macrophage subsets, we adopted a standardised protocol that reliably generates THP-1 derived M1 and M2 macrophages with molecular features that mimic human monocyte derived macrophages (27). A key feature of this protocol is the utilisation of three distinct culture stages (PMA priming, rest, and cytokine differentiation) to generate cultures enriched for M0, M1 and M2 macrophages (**Supplementary Figure 1A**). Importantly, this protocol enabled us to investigate at which stage/s in macrophage differentiation PHX might act. As reported, these macrophage subsets had distinct morphological features (**Supplementary Figure 1B**). Flow cytometric measurement of M1 (CD80) and M2 (CD209) cell surface markers confirmed that the polarizing conditions generated the expected macrophage subsets (**Supplementary Figures 1C, D**). Furthermore, viability was high following culture, although higher in M2 than M1 cultures (**Supplementary Figure 1E**). This may reflect the fact that LPS, a key inducing factor in M1 macrophage differentiation, is cytotoxic to macrophages. Reported LPS concentrations in M1 cultures ranges from 0.01 to 1 μg/mL (40). We titrated the LPS concentration used in our M1 cultures and showed that 250 ng/mL LPS was required to induce a homogenous M1 macrophage population (**Supplementary Figures 2A, B**). Furthermore, lowering the LPS concentration reduced the CD80 expression per cell (as measured by mean fluorescence intensity (MFI)) (**Supplementary Figure 2C**). Thus, we used 250 ng/mL LPS for all subsequent M1 cultures as in the previously published protocol (27).

**Figure 1:**
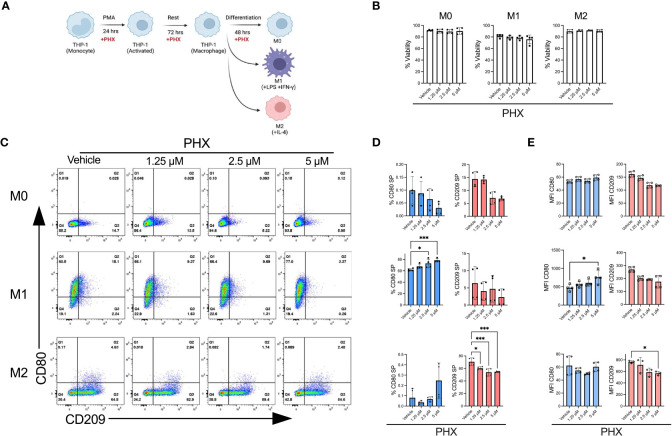
Perhexiline (PHX) promotes THP-1 monocyte differentiation into M1 macrophages. **(A)** Schematic diagram showing PHX addition during PMA mediated THP-1 M0, M1 and M2 differentiation. **(B)** Viability of THP-1 differentiated macrophages with or without PHX treatment. **(C)** Flow cytometry plots showing the expression of CD80 and CD209 for THP-1 differentiated macrophages with or without PHX treatment. **(D)** Quantification of % CD80 (blue) single positive (SP, Q1 gate) and % CD209 (pink) SP (Q3 gate) with or without PHX treatment and **(E)** MFI values of CD80 and CD209 of THP-1 differentiated macrophages (M0 top, M1 middle, M2 bottom). Data are pooled from two independent experiments with duplicates (n=4) and shown as mean ± SD. One-way ANOVA with Dunnett's multiple comparisons test was performed for the analysis *p ≤ 0.05; ***p ≤ 0.001.

Upon optimising our macrophage culture conditions, we investigated if PHX affected M1 or M2 macrophage polarization. As PHX is known to have cytotoxic activity, we first determined that THP-1 cells tolerated up to 5 µM PHX for 72 hours with no signs of toxicity, as shown by the alamarBlue viability assay (data not shown). Next, we determined the effect of 1.25, 2.5 and 5 µM PHX on the M0, M1 and M2 cultures (**Figure 1A**). Addition of PHX did not significantly reduce the viability in any of the cultures (**Figure 1B**). By itself PHX did not promote polarization of M0 cells into either M1 or M2 macrophages. We observed a PHX concentration dependent increase in the proportion of M1 macrophages in the M1 cultures, and a decrease in M2 macrophages in the M2 cultures (**Figures 1C–E**). Furthermore, the reduction in M2 macrophages was not the result of a shift to M1 macrophage polarization in the M2 culture. These data suggests that PHX treatment inhibited M2 macrophage differentiation, and enhanced M1 macrophage differentiation only in conjunction with M1 promoting factors such as LPS and IFN-γ.

### PHX-mediated M1 polarization occurs during the differentiation stage

**Figure 2:**
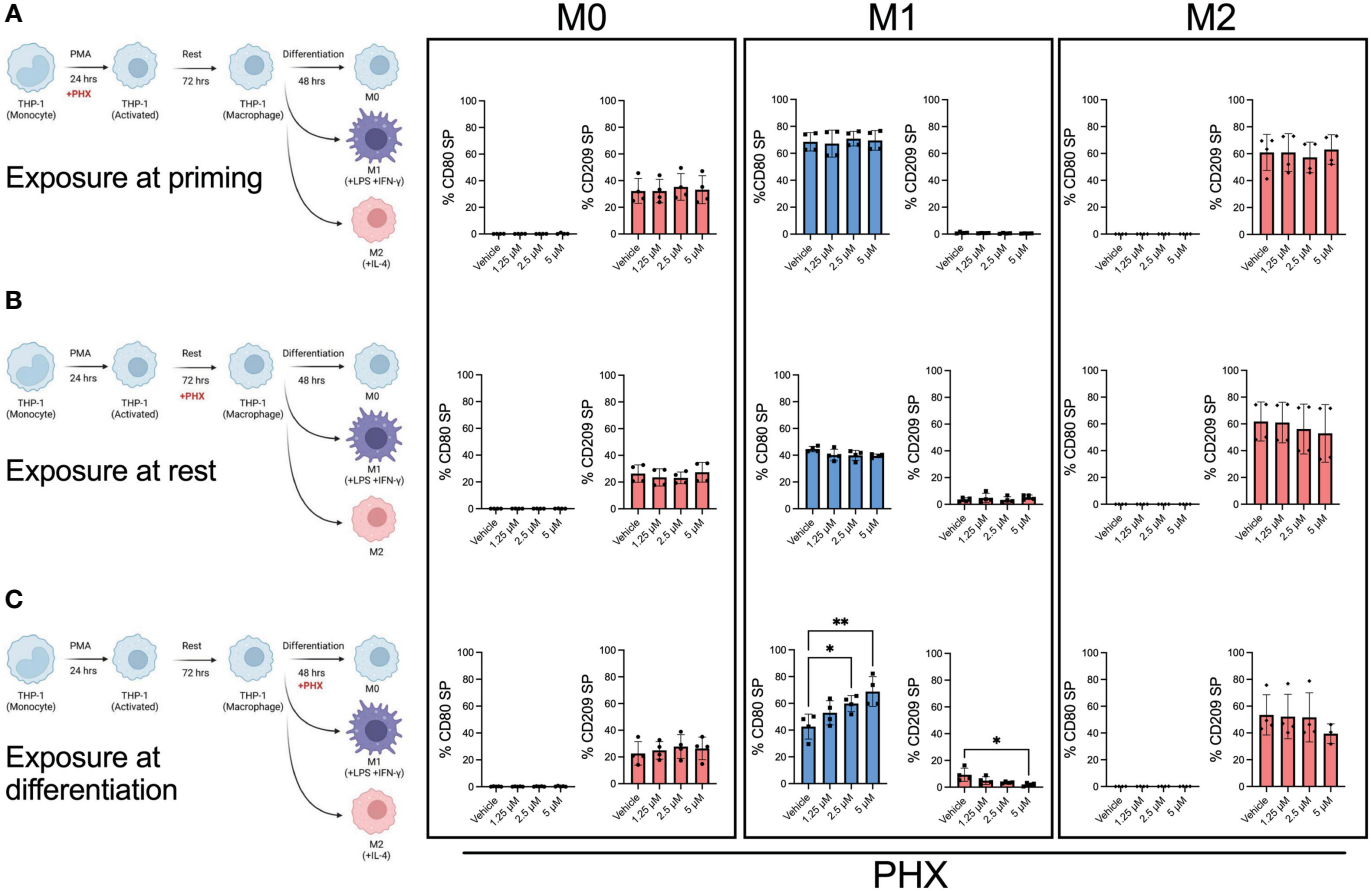
M1 promotion by PHX occurs during the differentiation stage. PHX was added at either **(A)** priming, **(B)** resting or **(C)** differentiation stage of the macrophage culture. Schematic diagram describing where PHX was added and quantification of % CD80 (blue) single positive (SP) and % CD209 (pink) SP between M0, M1 and M2 differentiation conditions. Data are pooled from two independent experiments with duplicates and presented as mean ± SD of pooled replicates. One-way ANOVA with Dunnett's multiple comparisons test was performed for the analysis *p ≤ 0.05; **p ≤ 0.01.

**Figure 3:**
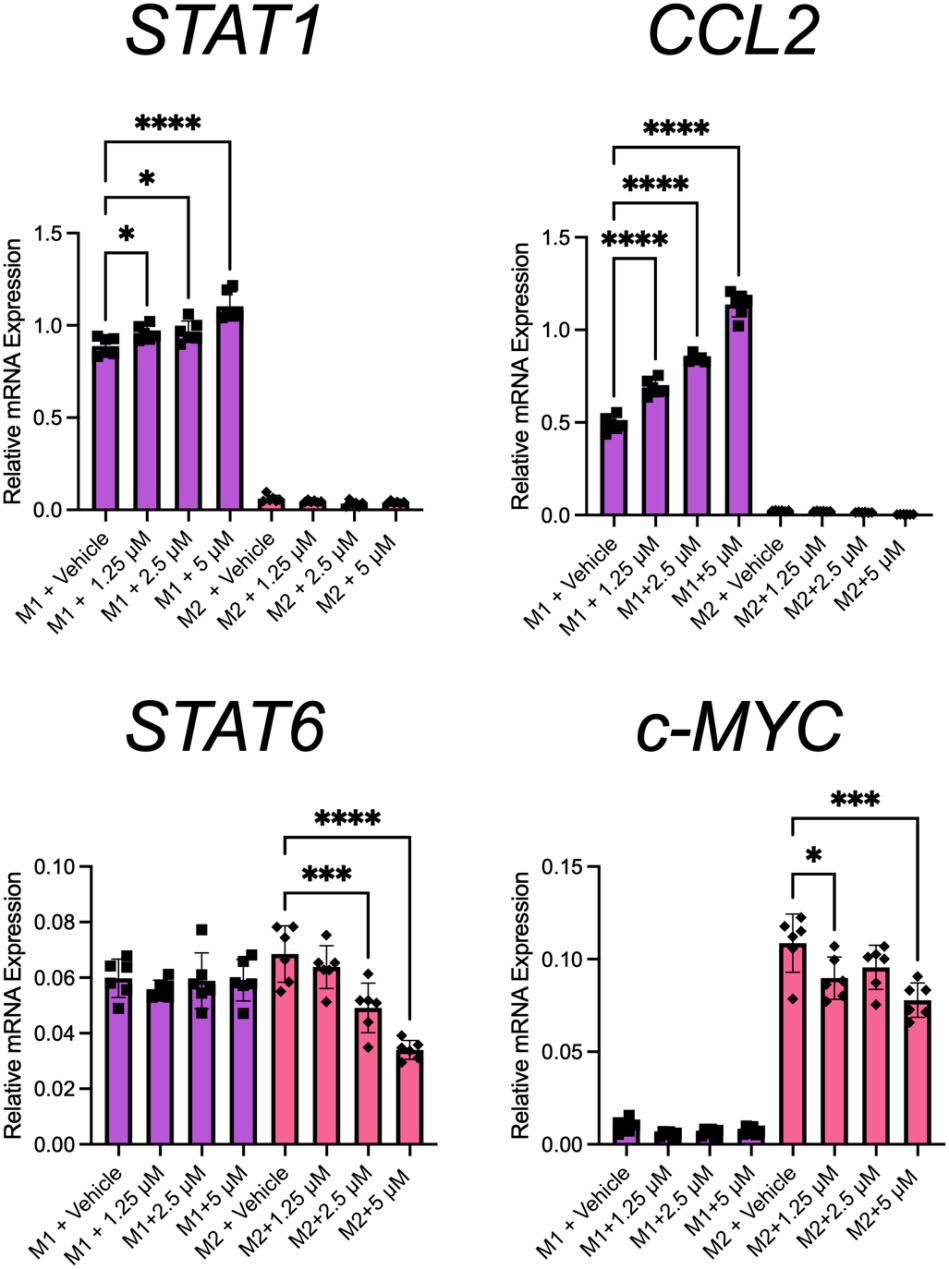
PHX promotes M1 macrophage transcriptional programming. THP-1 derived M1 or M2 macrophages were treated with PHX during their differentiation stage and qRT-PCR. mRNA expression of *STAT1*, *CCL2*, *STAT6* and *c-MYC* in M1 (purple) and M2 (pink) macrophages. Data are pooled from 2 independent experiments with 3 replicates from each experiment and presented as mean ± SD of pooled replicates. One-way ANOVA with Dunnett's multiple comparisons test was performed for the analysis *p ≤ 0.05; ***p ≤ 0.001; ****p ≤ 0.0001.

To determine at which stage PHX had its effect, we selectively added it or vehicle at either the PMA priming, rest, or differentiation stage of the M0, M1, and M2 cultures (**Figure 2**). Our results showed that the addition of PHX specifically at the differentiation stage of the M1 cultures was enough to enhance M1 macrophage polarization. We observed a significant increase in CD80^+^ M1 macrophages and a decrease in the already low count of CD209+ M2 macrophages present in our M1 cultures (**Figure 2C**). In support, PHX treatment at the differentiation stage in the M1 cultures significantly increased expression of key M1 macrophage genes *STAT1* and *CCL2* (**Figure 3**). In contrast, we did not detect a PHX induced reduction in CD209+ M2 macrophages at any of the stages in the M2 cultures (**Figure 2**). However, we did observe a significant decrease in M2 macrophage related transcription factors *STAT6* and c-*MYC* when PHX was added during the cytokine differentiation stage of the M2 cultures (**Figure 3A**). This suggests that PHX may have partially inhibited M2 macrophage polarization, but higher concentrations or longer treatment times are required to induce a strong inhibitory effect as observed in **Figure 1**. Taken together, our results suggest that PHX may enhance M1 and inhibit M2 macrophage polarization. Furthermore, PHX acts primarily when activated THP-1 macrophages are exposed to M1 differentiating factors.

### Analysis of differentially enriched proteins in PHX-treated M1 macrophage cultures

Because the effect of PHX was most pronounced in M1 cultures, we next characterised the changes it induced in the proteome of M1 macrophages (**Supplementary Figure 3A**). Five experimental replicates of vehicle and PHX treated (at the cytokine differentiation stage) THP-1 M1 cultures were subjected to DIA MS, a technique that allows for reproducible quantitative deep proteome-wide profiling (41, 42). Each treatment showed high homogeneity between replicates (**Supplementary Figures 3B, C**). We identified a total of 2,999 unique proteins, with 62 and 15 proteins found exclusively in vehicle and PHX treated M1 cultures, respectively (**Supplementary Figure 3D**; **Table S1**). Interestingly, PHX treatment induced significant changes to the M1 macrophage proteome (**Supplementary Figure 3E**).

**Figure 4:**
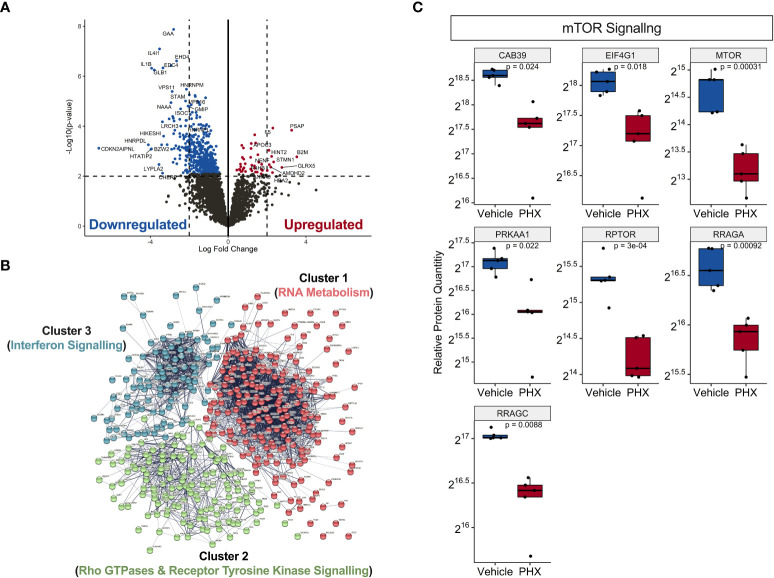
Proteomic analysis on PHX treated M1 macrophages reveal downregulation in immune signalling and RNA metabolism. **(A)** Volcano plot showing statistically significant upregulated (red) and downregulated (blue) proteins in 5 μM PHX treated M1 macrophages. **(B)** Network analysis based on protein-protein interactions (PPI) of 433 significantly downregulated proteins in PHX treated M1 macrophages. The k-means clustering identified 3 clusters with distinct Reactome Knwoledgbase pathway associated proteins. **(C)** Box plot comparing relative protein expression of identified mTOR signalling proteins between vehicle or PHX treated M1 macrophages. Student’s unpaired t-tests.

Using differential expression analysis of quantitative mass spectrometry data (DEqMS) (28), we identified 488 differentially expressed proteins (DEP, adj. p-value <0.05) with annotated gene names that were altered by PHX treatment (**Figure 4A**). Of these, 44 proteins were upregulated and 444 were downregulated (**Table S2**), and 11 upregulated and 80 downregulated proteins had at least a 2-log fold change in expression (**Figure 4A**; **Table 1**). Using the lists of upregulated and downregulated DEPs, we used the STRING database to visualise the protein-protein interaction (PPI) networks that were affected by PHX treatment (30). We identified that the 433 downregulated DEPs generated a PPI network consisting of 3 main clusters (**Figure 4B**; **Table S3**). Gene set enrichment analysis (GSEA), based on the Reactome Knowledgebase (31), showed that Cluster 1 proteins were primarily involved in RNA metabolism and could be subdivided into RNA splicing and protein translation (**Supplementary Figure 4A**; **Table S4**). Clusters 2 and 3 had proteins primarily related to the immune system, with Cluster 3 consisting of interferon signalling proteins (**Supplementary Figure 4**; **Table S4**). Cluster 2 contained diverse signalling pathways including Rho GTPase, mTOR, and Receptor Tyrosine Kinase (RTK). RTKs could be attributed to receptors for growth factors, cytokines, and hormones which in Cluster 2 included VEGFR2, MET and Eph receptors (**Supplementary Figure 4B**). Since PHX has been shown to downregulate mTOR signalling (43, 44), we confirmed that PHX downregulated mTOR, raptor, and other mTOR related proteins in M1 cultures (**Figure 4C**). In contrast, the 44 upregulated DEPs did not form any strong PPI networks (data not shown).

**Table 1:** Significantly (> 2 logFC) expressed proteins in PHX treated M1 macrophages.

Gene	logFC	Adj. p-value	Gene	logFC	Adj. p-value
**B2M**	3.529495929	0.023052796	**DOCK4**	-2.258267991	0.017048292
**PSAP**	3.26357242	0.008276641	**CDC123**	-2.261883311	0.01173899
**GLRX5**	2.761869289	0.036763963	**ARMC6**	-2.282290738	0.012187523
**STMN1**	2.337948587	0.028754691	**MAP3K7IP1**	-2.310405241	0.054414936
**F5**	2.297849597	0.007455963	**AGA**	-2.315869961	0.01123942
**ANXA6**	2.2789641	0.047294602	**SGSH**	-2.362656867	0.015316252
**HINT2**	2.23403248	0.02255069	**LRCH3**	-2.43772221	0.007536253
**AMDHD2**	2.190221643	0.035827761	**ISOC1**	-2.444095831	0.002738254
**NENF**	2.113458013	0.031830485	**INPP4A**	-2.473736427	0.010679874
**APOC3**	2.095560484	0.016795917	**HCK**	-2.475294822	0.019045496
**P4HB**	2.010677452	0.043076785	**SH3GL1**	-2.480084459	0.021608381
**HNRNPC**	-2.000486188	0.043041306	**BCL2L13**	-2.484614354	0.027827444
**GMIP**	-2.002062187	0.005103242	**EYA3**	-2.487509912	0.005103242
**RAB6A**	-2.019343724	0.018858523	**TTC39C**	-2.487884616	0.008880701
**EIF2S2**	-2.021086712	0.015816813	**SGK3**	-2.519217744	0.015816813
**BCAR1**	-2.024191182	0.010679874	**ASNS**	-2.580818664	0.01830534
**RALY**	-2.038142206	0.027671556	**WDR11**	-2.638487828	0.037281486
**N-PAC**	-2.044205898	0.018215012	**EHD4**	-2.65181695	0.000420415
**UBA5**	-2.044497294	0.015816813	**PRKACB**	-2.689272182	0.016454835
**DYNC1LI2**	-2.060187893	0.050485301	**RILPL2**	-2.732718704	0.029028551
**VPS16**	-2.060391633	0.003791233	**WASH6P**	-2.740885086	0.008049586
**RPL13**	-2.0857813	0.021608381	**SF3B4**	-2.773714899	0.018497889
**CMAS**	-2.096780554	0.012187523	**CASP4**	-2.781069256	0.005943747
**CDK2**	-2.099718493	0.053841326	**STAM2**	-2.803073835	0.01173899
**MSTO1**	-2.101065107	0.021425515	**GAA**	-2.804421997	0.000278774
**FRG1**	-2.110086015	0.015816813	**SCPEP1**	-2.81834001	0.007571394
**LEO1**	-2.126320592	0.023933753	**TOE1**	-2.829288808	0.032234822
**DPYSL4**	-2.136251638	0.04551332	**EHD1**	-2.849938981	0.007973589
**TRADD**	-2.137198805	0.012187523	**VPS11**	-2.879644803	0.000420415
**NCOA7**	-2.142719702	0.004417789	**SKIV2L**	-2.908928488	0.019186401
**PRCP**	-2.142994149	0.051384751	**EDC4**	-2.935160217	0.000420415
**GLA**	-2.144514267	0.021608381	**NAAA**	-2.938934439	0.002738254
**HNRNPM**	-2.144645723	0.002738254	**APOL2**	-3.032731763	0.005826693
**VPS45**	-2.160128184	0.015816813	**FUBP1**	-3.137140732	0.015816813
**INPP5B**	-2.160437446	0.023508086	**HIKESHI**	-3.318343484	0.012187523
**CCNK**	-2.162951069	0.016989682	**GLB1**	-3.349477293	0.000577327
**RPL29**	-2.165546676	0.029180256	**CHERP**	-3.372428857	0.058518182
**PIK3AP1**	-2.169278394	0.008662874	**HSP90AA4P**	-3.378181329	0.007571394
**SEMA4A**	-2.174210933	0.015816813	**SYNE1**	-3.422012064	0.016336037
**SMC2L1**	-2.180532646	0.028617701	**IL4I1**	-3.527897787	0.000278774
**PEF1**	-2.188540159	0.011462339	**LYPLA2**	-3.555869354	0.038923739
**STAM**	-2.190570048	0.002738254	**IL1B**	-3.93575407	0.000577327
**NUDT4**	-2.191147736	0.03749938	**BZW2**	-3.942261934	0.02030524
**IFITM1**	-2.192359261	0.012187523	**HTATIP2**	-3.962754975	0.019692511
**HNRNPL**	-2.192565806	0.01173899	**HNRPDL**	-4.103793986	0.018468561
**IFT27**	-2.199539218	0.048952426	**CDKN2AIPNL**	-6.649652917	0.021122993

Bold was to easily identify the gene name. The highlight red represents proteins that were upregulated and blue were downregulated proteins.

**Figure 5:**
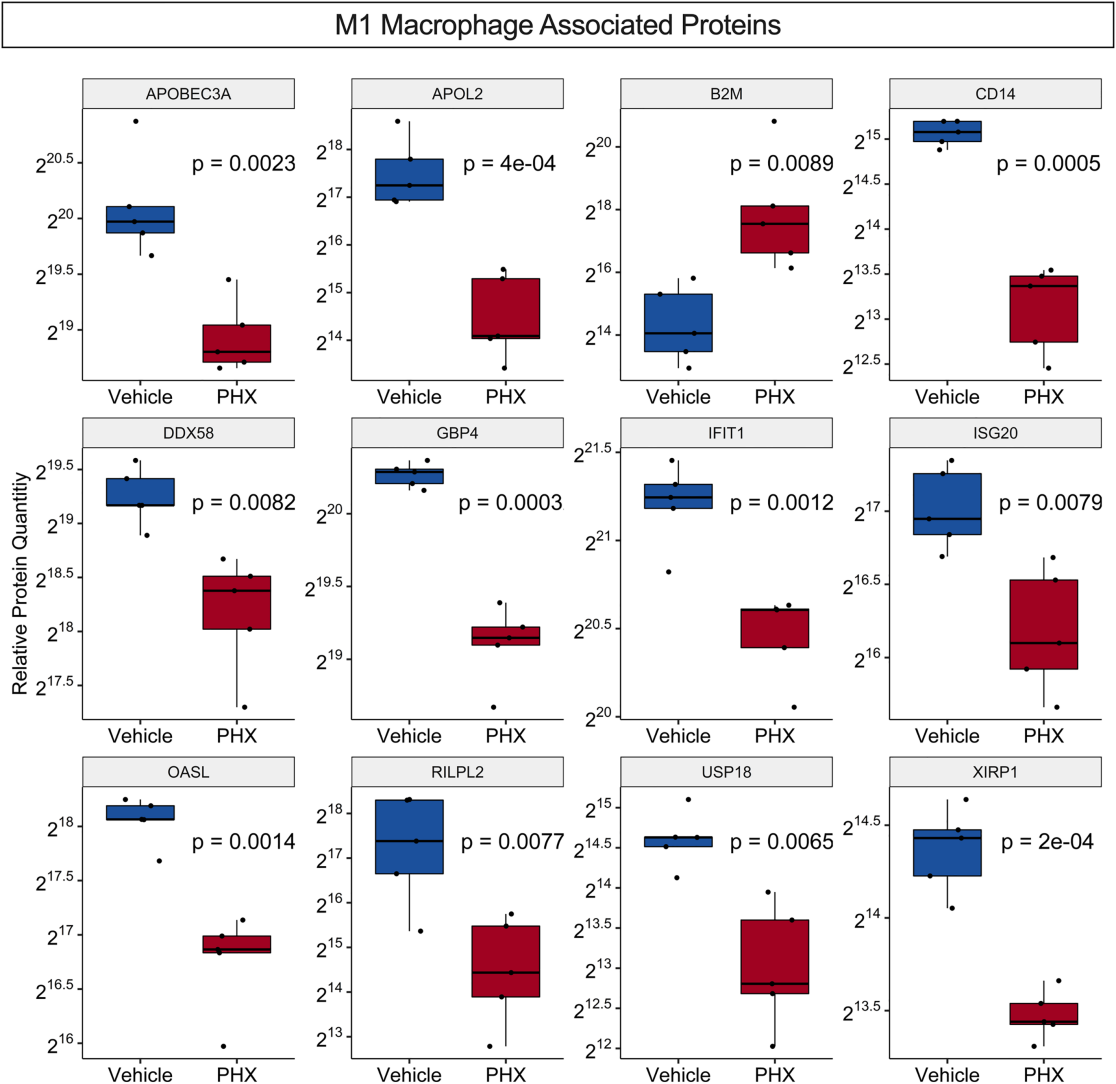
PHX alters expression of M1 macrophage associated proteins. Box plot comparing relative protein expression of known unique THP-1 M1 macrophage specific proteins between vehicle or 5 μM PHX treated M1 macrophages. Student’s unpaired t-tests.

Recently a proteomic analysis of THP-1 derived M0, M1 and M2 macrophage cultures identified 68 M1-associated proteins and 20 M2-associated proteins that were found only in their respective cultures (**Table S5**) (45). While our culture conditions do not exactly match that study, comparisons of the two data sets identified 33 M1- and three M2-associated proteins in common, supporting that our M1 culture conditions promote differentiation of M1 macrophage phenotypes (**Table S6**). DEqMS for the identified 33 M1-associated proteins in common showed that PHX treatment significantly downregulated 11 M1-associated proteins and upregulated one, B2M (**Figure 5**). Thus, PHX treatment while promoting M1 macrophage polarization may eventually downregulate the inflammatory properties of these cells.

### GSEA of PHX-treated M1 macrophage cultures

**Figure 6:**
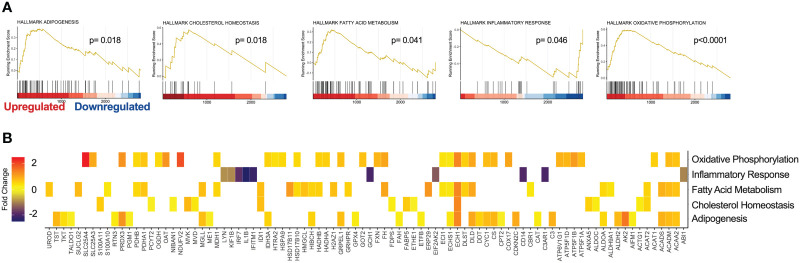
Gene Set Enrichment Analysis (GSEA) reveals cellular pathways modified by PHX on M1 macrophages. GSEA of 2999 proteins derived from vehicle or 5 μM treated M1 macrophages using the Molecular Signatures Database (MSigDB) Hallmark data set. **(A)** Enrichment Score (ES) plots of statistically significant enriched Hallmark data gene sets. **(B)** Heatmap identifying protein expression associated with each Hallmark gene set.

To gain a deeper insight into the global changes within our proteomics data, pathway enrichment analysis was performed on all 2999 unique proteins identified by DIA MS. Using the MSigDB, we performed GSEA against Hallmark, Reactome Knowledgebase and Gene Ontology (GO) datasets (35, 36). Interestingly, PHX treatment significantly enriched Hallmark gene sets for oxidative phosphorylation, adipogenesis, cholesterol homeostasis, and fatty acid metabolism. Furthermore, in agreement with our differential enrichment analysis, PHX downregulated genes related to the inflammatory response (**Figure 6**; **Table S7**). Reactome Knowledgebase pathway analysis supports these findings, with key pathways in the TCA cycle, mitochondrial fatty acid beta oxidation, and gluconeogenesis being upregulated (**Supplementary Figure 5**; **Table S8**). Finally, our GO Cellular Component analysis shows that PHX upregulated proteins related to the cytoplasm, mitochondria and cell membrane, and downregulated proteins related to the nucleus and endosome (**Figure S6**; **Table S9**). This once again matches our GO Biological Process (**Supplementary Figure 7**; **Table S10**) and GO Molecular Function (**Supplementary Figure 8**; **Table S11**), where there is an enrichment for metabolic process related to fatty acid metabolism and oxidative phosphorylation with a downregulation in RNA metabolism.

### Validation of proteomic analyses

**Figure 7:**
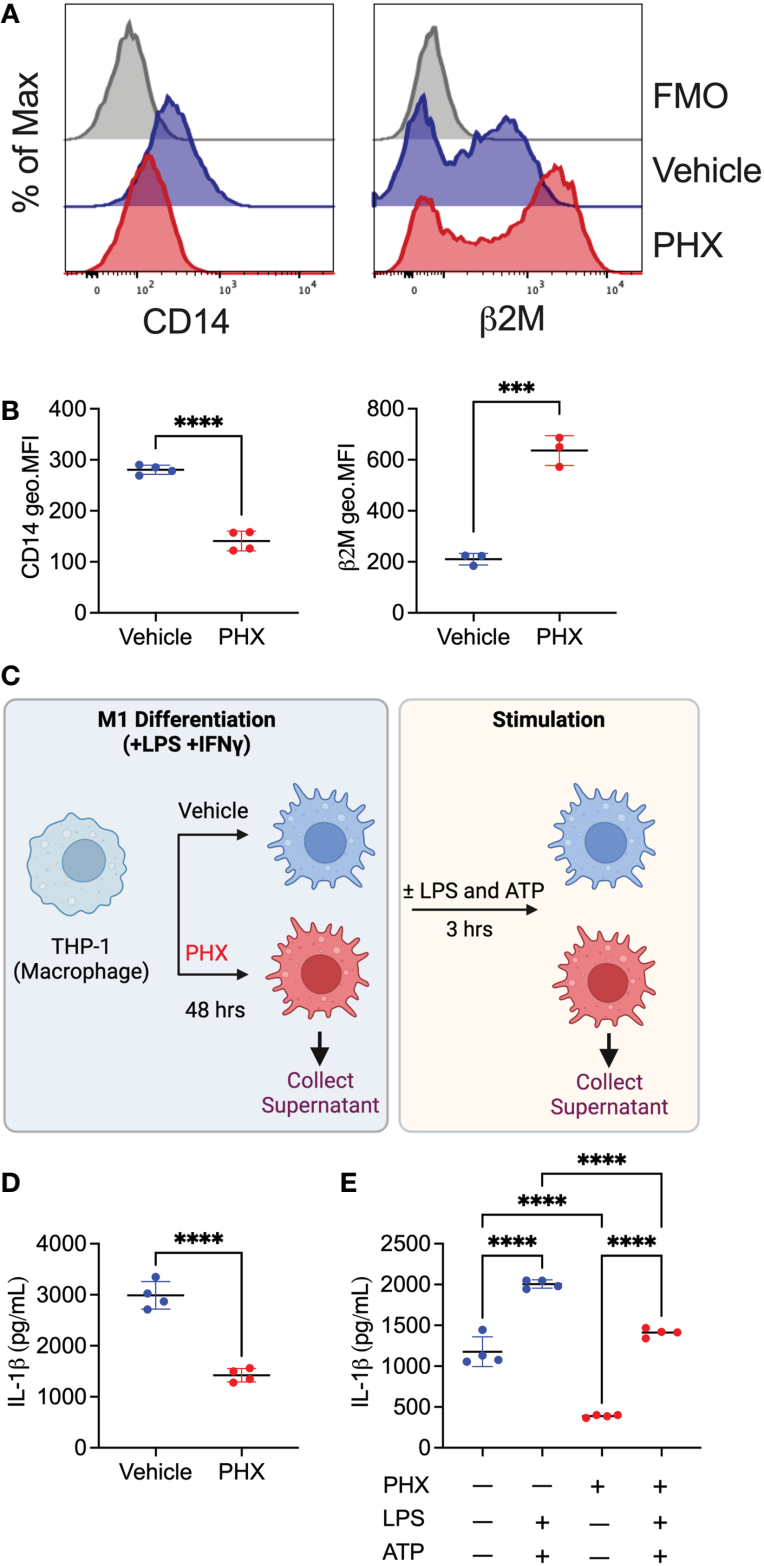
Validation of proteomics data set. THP-1 derived M1 macrophages cultured with 5 μM PHX or vehicle control were examined for changes in expression of proteins by flow cytometry or ELISA. **(A)** Histogram plots and **(B)** quantitation of geometric mean fluorescence intensity (geo.MFI) for the expression of CD14 and β2M on CD80+ macrophages. **(C)** Experimental workflow describing the collection of supernatants for IL-1β detection. Supernatants **(D)** prior to stimulation or **(E)** after stimulation with LPS and ATP was collected from PHX-treated M1 macrophages. Data are pooled 3-4 replicates and presented as mean ± SD. Student’s t-test or One-way ANOVA with Dunnett's multiple comparisons test was performed for the analysis. ***p ≤ 0.001; ****p ≤ 0.0001.

To validate our proteomics result, we assessed the cell surface expression of CD14 and B2M on PHX-treated M1 macrophages by flow cytometry. Consistent with our proteomics results (**Figure 5**), PHX treatment of M1 macrophage cultures resulted in reduced expression of CD14 and enhanced expression of B2M (**Figures 7A, B**). Finally, as IL-1β was one of the most significantly downregulated proteins in our data (**Figures 4A**, **6B**), we performed ELISA to investigate if PHX reduced IL-1β release in our M1 cultures (**Figure 7C**). Cell culture supernatants from PHX-treated M1 cultures had lower IL-1β levels compared to vehicle controls (**Figure 7D**). Furthermore, stimulation of these M1 cultures with LPS and ATP showed that while PHX treatment reduced the amount of IL-1β released, it did not prevent these macrophages from secreting IL-1β (**Figure 7E**). Taken together, for the first time, we define the PHX induced changes in the M1 macrophage proteome.

## Discussion

Macrophage dysregulation is common in a range of diseases. For example, an increase in M1-like macrophages is associated with the chronic inflammation (6), while an increase in M2-like macrophages in cancer is associated with the suppression of anti-tumour immune responses, angiogenesis, cancer outgrowth and metastasis (7, 8). The many findings of associations between altered macrophage states and disease suggests that drugs which alter macrophage activation and polarisation may have potential in the treatment of such diseases. Here we report that PHX treatment inhibited M2 and promoted M1 macrophage differentiation in our polarisation cultures, and that proteomic analysis suggests that macrophages in PHX treated M1 cultures may have adopted an ‘immune suppressed’ state. Our findings show that PHX may have underappreciated effects on macrophage biology.

The drug PHX has been used clinically for decades in the treatment of cardiovascular disease, where it is thought to act by reducing FAO through the inhibition of CPT1. This shifts myocardial energy generation away from fatty acid toward a greater reliance on carbohydrate metabolism, which maintains myocardial production of ATP but with a lower oxygen utilisation. We and others have reported that PHX is cytotoxic to cancer cells *in vitro* and *in vivo*. The underlying mechanisms for the effects on cancer cells remain unclear, with reports suggesting that several cell pathways may be involved, not just the inhibition of CPT1. It is not clear if these other pathways reported to be altered by PHX in cancer cells are specific primary targets or are activated as a downstream effect of its binding to CPT1 or are a consequence of cell damage or death resulting from the drug. Because of its inhibitory effects on cancer cells in a number of experimental models, there is interest in whether PHX has the potential to be repurposed as an adjunctive therapeutic in the management of cancer (46–48).

The growth of a cancer is dependent on more than just the properties of the cancer cells. Carcinoma establishment and progression, including invasion and metastasis, is greatly influenced by interactions between epithelial or tumour cells and local stromal elements. Macrophages are an important functional cell within the tumour microenvironment and can positively or negatively influence tumour progression. Macrophages can be classified according to how they are activated or their function. Classically activated macrophages (M1) tend to inhibit tumour progression, while alternatively activated macrophages (M2) tend to promote the progression. The presence and activity of these subsets is not confined to tumours. The M1 macrophages in general are pro-inflammatory, while the M2 macrophages tend to dampen inflammation and promote tissue repair and growth. Imbalance of the ratio of M1/M2 cells has been reported to be associated with several diseases with an immune component, such as asthma, inflammatory bowel disease or fibrotic diseases. A novel therapeutic approach in such diseases could be the targeting of the macrophages with drugs which alter their phenotype away from that which promotes the disease. Thus, drugs which promote M1 macrophage differentiation may be useful to treat cancer or allergic diseases where overabundant type-2 inflammation orchestrated by M2 macrophages is associated with worse clinical outcomes (49).

Using standard protocols to differentiate the human THP-1 cell line into macrophages (27), we have shown that the addition of PHX to M1 macrophage cultures resulted in more M1-like macrophages. Our findings are consistent with a recent report that PHX could shift murine bone marrow-derived M2 macrophages to an M1 macrophage profile (24) and that PHX delivery *in vivo* can suppress M2 polarisation in a mouse model of chronic kidney disease (50). However, our proteomic analysis of PHX treated M1 macrophages showed that in the treated cells, key proteins related to inflammation were downregulated. Importantly we observed the downregulation of IL-1β, a key secreted protein for macrophage-mediated inflammation (51), both in the cell lysate (proteomics) and culture supernatant (ELISA). This immunosuppressive effect has been observed in mouse models of sepsis and atherosclerosis where PHX treatment inhibited inflammatory macrophage responses including IL-1β through the activation of KLF14 (25, 26).

Our DIA MS quantitative proteomics analysis provides important insights into the effects of PHX on M1 macrophages. We identified 488 differentially expressed proteins altered by PHX treatment of these differentiating macrophages. Gene set enrichment analysis showed that in the M1 macrophages the main protein networks which were affected by PHX were associated with RNA metabolism, particularly related to RNA translation, and immune responses. It was interesting to observe that PHX downregulated proteins related to interferon signalling, a key component of M1 macrophage activation and function. Relevant to this, we identified an immune response protein network cluster representing Rho-GTPases, receptor tyrosine kinases (RTKs) and mTOR signalling. Previous studies have reported that PHX can mediate inhibition of mTOR, which is involved in regulation of cell growth and metabolism (43, 44). mTOR is found in two distinct molecular complexes, mTORC1 and mTORC2, which differ structurally and functionally. Characterization of mTOR related proteins suggest that PHX inhibited mTORC1 signalling as evidenced by downregulation of key proteins RPTOR, RRAGA, RRAGC, and EIF4G1. In macrophages, mTORC1 signalling is associated with M1 differentiation and activation. Loss of mTORC1 signalling has been shown to enhance M1 macrophage function *in vivo* (52).

RTKs represent a broad range of receptors involved in a variety of cellular responses. PHX disrupted downstream RTK proteins found in the VEGFR2, MET and Eph receptor signalling pathways. VEGFR2 signalling promotes immunosuppressive functions in myeloid cells and M2 macrophages including the upregulation of PD-L1 (53, 54). MET promotes the switching of M1 phenotype to M2 phenotype (55) and dampens M1 cytokine production such as IL-6 (56). Eph receptors are involved in macrophage adhesion and migration but there is little evidence that they affect polarization (57). Rho-GTPases are involved in macrophage motility and phagocytosis (58–60). We did not observe changes to key effector proteins in this pathway and most proteins identified are shared with RTK signalling. Together these data suggest that PHX downregulates a spectrum of proteins involved in M1 macrophage polarization and function. Indeed, when we measured protein levels of M1 macrophage unique proteins, all but B2M were downregulated with PHX treatment.

Of the few proteins upregulated, only B2M and PSAP were increased by at least 3 log fold. B2M, beta-2-microglobulin, is a critical component of MHC class I complex and is critical for immune activation and has anti-bacterial properties when secreted (61). PSAP, prosaposin, is a lysosomal protein involved in glycosphingolipid metabolism, and has recently been shown to promote glycolysis and oxidative phosphorylation in macrophages and is inhibited by mTOR signalling (62). Thus, inhibition of mTOR by PHX may have resulted in upregulation of PSAP in the M1 cultures.

Further investigations are required to determine the relationship between the observed changes in protein levels and functional consequences. PHX is a reported inhibitor of CPT1, a key rate-limiting enzyme involved in mitochondrial FAO by transporting fatty acids into the mitochondria (22). In our GSEA analysis, it was interesting to see an enrichment and upregulation of proteins associated with oxidative phosphorylation, fatty acid metabolism, adipogenesis and cholesterol homeostasis identified using the MSigDB hallmark gene set. Consistent with this finding, one of the first studies describing PHX-mediated oxidative phosphorylation and fatty acid oxidation inhibition also showed that inhibition leads to the accumulation of proteins related to complex 1 and II of the respiratory chain in hepatocytes (63). Importantly, Oyarce et al. has demonstrated that PHX treatment impaired oxidative phosphorylation including mitochondrial basal and maximal respiration in M2 macrophages (24). While we also saw a statistically significant enrichment of cholesterol homeostasis and adipogenesis gene sets, careful interpretation of this data is required as most proteins identified were shared in oxidative phosphorylation and fatty acid metabolism, hence detection of these pathways in our GSEA analysis may be in part due to an accumulation of proteins shared amongst these pathways. Nevertheless, follow-up studies including functional metabolic assays are required to confirm whether the increases observed for mitochondrial proteins in response to PHX treatment correspond to compensatory changes, maintenance of cellular homeostasis, or reflect a metabolic perturbation induced by PHX treatment. The consequences of this putative metabolic perturbation, for example in energy-intensive functions of macrophages such as motility or phagocytosis, remain to be investigated and may have implications for how PHX might affect macrophage function *in vivo*. It was recently shown that M2 macrophage reprogramming to M1 macrophages is possible by using metabolic drug targets including PHX in mice (24). Future studies are also required to analyse the effects of PHX treatment on human M2 macrophage repolarisation and subsequent function. We have preliminary data to suggest that PHX can enhance M2 repolarisation to M1 in the presence of LPS (data not shown). Finally, it would also be valuable to know if the proteomic changes reported here are replicated *in vivo* by studying different macrophage-mediated *in vivo* disease models. PHX has been reported to influence macrophage function in mouse models of sepsis and kidney disease (26, 50).

In this report, we show that PHX treatment inhibits the differentiation of THP-1 cells into M2 macrophages and promotes their differentiation into M1 macrophages with altered expression of inflammatory pathways. We publish two novel datasets: the baseline proteome of M1 macrophage cultures generated using a standardised published protocol for THP-1 cells (27), and the proteomic changes induced by PHX treatment of M1 macrophages. We envision that this resource will be useful in future studies into the effect of PHX in macrophage-related diseases.

## Data availability statement

The datasets presented in this study can be found in online repositories. The names of the repository/repositories and accession number(s) can be found in the article/**Supplementary Material**.

## Author contributions

Conceptualisation KF, BD; methodology, investigation, and data analysis, BD, CML, RL, ES, KF, AC, NC, TC, MR, GH, GB; resources GM, KF, BS, writing—original draft preparation, BD, and KF. writing—review and editing, PD, ES, KF, BS; supervision, GM, GL, PD, KF, SV; funding acquisition, BD, GM, KF. All authors contributed to the article and approved the submitted version.

## References

[1] Murray PJ Wynn TA. Protective and pathogenic functions of macrophage subsets. Nat Rev Immunol (2011) 11(11):723–37. doi: 10.1038/nri3073 PMC3422549 21997792

[2] Mosser DM Edwards JP. Exploring the full spectrum of macrophage activation. Nat Rev Immunol (2008) 8(12):958–69. doi: 10.1038/nri2448 PMC2724991 19029990

[3] Ma RY Black A Qian BZ. Macrophage diversity in cancer revisited in the era of single-cell omics. Trends Immunol (2022) 43(7):546–63. doi: 10.1016/j.it.2022.04.008 35690521

[4] Locati M Curtale G Mantovani A. Diversity, mechanisms and significance of macrophage plasticity. Annu Rev Pathol (2020) 15:123–47. doi: 10.1146/annurev-pathmechdis-012418-012718 PMC7176483 31530089

[5] Kadomoto S Izumi K Mizokami A. Macrophage polarity and disease control. Int J Mol Sci (2022) 23(1):144. doi: 10.3390/ijms23010144 PMC8745226 35008577

[6] Funes SC Rios M Escobar-Vera J Kalergis AM. Implications of macrophage polarization in autoimmunity. Immunology (2018) 154(2):186–95. doi: 10.1111/imm.12910 PMC5980179 29455468

[7] Poh AR Ernst M. Targeting macrophages in cancer: From bench to bedside. Front Oncol (2018) 8:49. doi: 10.3389/fonc.2018.00049 29594035 PMC5858529

[8] Duan Z Luo Y. Targeting macrophages in cancer immunotherapy. Sig Transduct Target Ther (2021) 6(1):1–21. doi: 10.1038/s41392-021-00506-6 PMC7994399 33767177

[9] Barcik W Boutin RCT Sokolowska M Finlay BB. The role of lung and gut microbiota in the pathology of asthma. Immunity (2020) 52(2):241–55. doi: 10.1016/j.immuni.2020.01.007 PMC7128389 32075727

[10] Fazlollahi M Lee TD Andrade J Oguntuyo K Chun Y Grishina G. The nasal microbiome in asthma. J Allergy Clin Immunol (2018) 142(3):834–843.e2. doi: 10.1016/j.jaci.2018.02.020 29518419 PMC6123291

[11] Psaltis AJ Mackenzie BW Cope EK Ramakrishnan VR. Unraveling the role of the microbiome in chronic rhinosinusitis. J Allergy Clin Immunol (2022) 149(5):P1513–21. doi: 10.1016/j.jaci.2022.02.022 PMC9354834 35300985

[12] Krysko O Holtappels G Zhang N Kubica M Deswarte K Derycke L. Alternatively activated macrophages and impaired phagocytosis of s. aureus chronic rhinosinusitis. Allergy (2011) 66(3):396–403. doi: 10.1111/j.1398-9995.2010.02498.x 20973804

[13] Yamaguchi M Zacharia J Laidlaw TM Balestrieri B. PLA2G5 regulates transglutaminase activity of human IL-4-activated M2 macrophages through PGE2 generation. J Leukoc Biol (2016) 100(1):131–41. doi: 10.1189/jlb.3A0815-372R PMC4946619 26936936

[14] Takabayashi T Kato A Peters AT Hulse KE Suh LA Carter R. Increased expression of factor XIII-a in patients with chronic rhinosinusitis with nasal polyps. J Allergy Clin Immunol (2013) 132(3):584–592.e4. doi: 10.1016/j.jaci.2013.02.003 23541322 PMC3737402

[15] Liang Z Zhang Q Thomas CM Chana KK Gibeon D Barnes PJ. Impaired macrophage phagocytosis of bacteria in severe asthma. Respir Res (2014) 15(1):72. doi: 10.1186/1465-9921-15-72 24972601 PMC4086996

[16] Simpson JL Gibson PG Yang IA Upham J James A Reynolds PN. Impaired macrophage phagocytosis in non-eosinophilic asthma. Clin Exp Allergy (2013) 43(1):29–35. doi: 10.1111/j.1365-2222.2012.04075.x 23278878

[17] Ardura JA Rackov G Izquierdo E Alonso V Gortazar AR Escribese MM. Targeting macrophages: Friends or foes in disease? Front Pharmacol (2019) 10:1255. doi: 10.3389/fphar.2019.01255 31708781 PMC6819424

[18] Ponzoni M Pastorino F Di Paolo D Perri P Brignole C. Targeting macrophages as a potential therapeutic intervention: Impact on inflammatory diseases and cancer. Int J Mol Sci (2018) 19(7):1953. doi: 10.3390/ijms19071953 29973487 PMC6073303

[19] Viola A Munari F Sánchez-Rodríguez R Scolaro T Castegna A. The metabolic signature of macrophage responses. Front Immunol (2019) 10:1462. doi: 10.3389/fimmu.2019.01462 31333642 PMC6618143

[20] Kolliniati O Ieronymaki E Vergadi E Tsatsanis C. Metabolic regulation of macrophage activation. JIN (2022) 14(1):51–68. doi: 10.1159/000516780 PMC8787535 34247159

[21] Unger SA Robinson MA Horowitz JD. Perhexiline improves symptomatic status in elderly patients with severe aortic stenosis. Aust N Z J Med (1997) 27(1):24–8. doi: 10.1111/j.1445-5994.1997.tb00909.x 9079249

[22] Ashrafian H Horowitz JD Frenneaux MP. Perhexiline. Cardiovasc Drug Rev (2007) 25(1):76–97. doi: 10.1111/j.1527-3466.2007.00006.x 17445089

[23] Lee L Campbell R Scheuermann-Freestone M Taylor R Gunaruwan P Williams L. Metabolic modulation with perhexiline in chronic heart failure. Circulation (2005) 112(21):3280–8. doi: 10.1161/CIRCULATIONAHA.105.551457 16301359

[24] Oyarce C Vizcaino-Castro A Chen S Boerma A Daemen T. Re-polarization of immunosuppressive macrophages to tumor-cytotoxic macrophages by repurposed metabolic drugs. Oncoimmunology (2021) 10(1):1898753. doi: 10.1080/2162402X.2021.1898753 33796407 PMC7971325

[25] Wang H Guo Y Lu H Luo Y Hu W Liang W. Krüppel-like factor 14 deletion in myeloid cells accelerates atherosclerotic lesion development. Cardiovasc Res (2022) 118(2):475–88. doi: 10.1093/cvr/cvab027 PMC8803076 33538785

[26] Yuan Y Fan G Liu Y Liu L Zhang T Liu P. The transcription factor KLF14 regulates macrophage glycolysis and immune function by inhibiting HK2 in sepsis. Cell Mol Immunol (2022) 19:504–15. doi: 10.1038/s41423-021-00806-5 34983946 PMC8976055

[27] Baxter EW Graham AE Re NA Carr IM Robinson JI Mackie SL. Standardized protocols for differentiation of THP-1 cells to macrophages with distinct M(IFNγ+LPS), M(IL-4) and M(IL-10) phenotypes. J Immunol Methods (2020) 478:112721. doi: 10.1016/j.jim.2019.112721 32033786

[28] Zhu Y Orre LM Zhou Tran Y Mermelekas G Johansson HJ Malyutina A. DEqMS: A method for accurate variance estimation in differential protein expression analysis. Mol Cell Proteomics (2020) 19(6):1047–57. doi: 10.1074/mcp.TIR119.001646 PMC7261819 32205417

[29] Li YF Radivojac P. Computational approaches to protein inference in shotgun proteomics. BMC Bioinf (2012) 13(16):S4. doi: 10.1186/1471-2105-13-S16-S4 PMC3489551 23176300

[30] Szklarczyk D Gable AL Lyon D Junge A Wyder S Huerta-Cepas J. STRING v11: Protein–protein association networks with increased coverage, supporting functional discovery in genome-wide experimental datasets. Nucleic Acids Res (2019) 47(Database issue):D607–13. doi: 10.1093/nar/gky1131 PMC6323986 30476243

[31] Gillespie M Jassal B Stephan R Milacic M Rothfels K Senff-Ribeiro A. The reactome pathway knowledgebase 2022. Nucleic Acids Res (2022) 50(D1):D687–92. doi: 10.1093/nar/gkab1028 PMC8689983 34788843

[32] Yu G Wang LG Han Y He QY. clusterProfiler: an r package for comparing biological themes among gene clusters. OMICS (2012) 16(5):284–7. doi: 10.1089/omi.2011.0118 PMC3339379 22455463

[33] Wu T Hu E Xu S Chen M Gou P Dai Z. clusterProfiler 4.0: A universal enrichment tool for interpreting omics data. The Innovation (2021) 2(3):100141. doi: 10.1016/j.xinn.2021.100141 PMC8454663 34557778

[34] Subramanian A Tamayo P Mootha VK Mukherjee S Ebert BL Gillette MA. Gene set enrichment analysis: A knowledge-based approach for interpreting genome-wide expression profiles. Proc Natl Acad Sci (2005) 102(43):15545–50. doi: 10.1073/pnas.0506580102 PMC1239896 16199517

[35] Liberzon A Subramanian A Pinchback R Thorvaldsdóttir H Tamayo P Mesirov JP. Molecular signatures database (MSigDB) 3.0. Bioinformatics (2011) 27(12):1739–40. doi: 10.1093/bioinformatics/btr260 PMC3106198 21546393

[36] Liberzon A Birger C Thorvaldsdóttir H Ghandi M Mesirov JP Tamayo P. The molecular signatures database (MSigDB) hallmark gene set collection. Cell Syst (2015) 1(6):417–25. doi: 10.1016/j.cels.2015.12.004 PMC4707969 26771021

[37] Gene Ontology Consortium. The gene ontology resource: Enriching a GOld mine. Nucleic Acids Res (2021) 49(D1):D325–34. doi: 10.1093/nar/gkaa1113 PMC7779012 33290552

[38] Ashburner M Ball CA Blake JA Botstein D Butler H Cherry JM. Gene ontology: Tool for the unification of biology. Nat Genet (2000) 25(1):25–9. doi: 10.1038/75556 PMC3037419 10802651

[39] Kanehisa M Furumichi M Sato Y Ishiguro-Watanabe M Tanabe M. KEGG: integrating viruses and cellular organisms. Nucleic Acids Res (2021) 49(D1):D545–51. doi: 10.1093/nar/gkaa970 PMC7779016 33125081

[40] Genin M Clement F Fattaccioli A Raes M Michiels C. M1 and M2 macrophages derived from THP-1 cells differentially modulate the response of cancer cells to etoposide. BMC Cancer (2015) 15(1):577. doi: 10.1186/s12885-015-1546-9 26253167 PMC4545815

[41] Searle BC Swearingen KE Barnes CA Schmidt T Gessulat S Küster B. Generating high quality libraries for DIA MS with empirically corrected peptide predictions. Nat Commun (2020) 11(1):1548. doi: 10.1038/s41467-020-15346-1 32214105 PMC7096433

[42] Pino LK Just SC MacCoss MJ Searle BC. Acquiring and analyzing data independent acquisition proteomics experiments without spectrum libraries. Mol Cell Proteomics (2020) 19(7):1088–103. doi: 10.1074/mcp.P119.001913 PMC7338082 32312845

[43] Balgi AD Fonseca BD Donohue E Tsang TCF Lajoie P Proud CG. Screen for chemical modulators of autophagy reveals novel therapeutic inhibitors of mTORC1 signaling. PloS One (2009) 4(9):e7124. doi: 10.1371/journal.pone.0007124 19771169 PMC2742736

[44] Rathore R Caldwell KE Schutt C Brashears CB Prudner BC Ehrhardt WR. Metabolic compensation activates pro-survival mTORC1 signaling upon 3-phosphoglycerate dehydrogenase inhibition in osteosarcoma. Cell Rep (2021) 34(4):108678. doi: 10.1016/j.celrep.2020.108678 33503424 PMC8552368

[45] Li P Hao Z Wu J Ma C Xu Y Li J. Comparative proteomic analysis of polarized human THP-1 and mouse RAW264.7 macrophages. Front Immunol (2021) 12. doi: 10.3389/fimmu.2021.700009 PMC8276023 34267761

[46] Dhakal B Li CMY Li R Yeo K Wright JA Gieniec KA. The antianginal drug perhexiline displays cytotoxicity against colorectal cancer cells *In vitro*: A potential for drug repurposing. Cancers (2022) 14(4):1043. doi: 10.3390/cancers14041043 35205791 PMC8869789

[47] Kant S Kesarwani P Guastella AR Kumar P Graham SF Buelow KL. Perhexiline demonstrates FYN-mediated antitumor activity in glioblastoma. Mol Cancer Ther (2020) 19(7):1415–22. doi: 10.1158/1535-7163.MCT-19-1047 PMC7335329 32430486

[48] Wang Y Lu JH Wang F Wang YN He MM Wu QN. Inhibition of fatty acid catabolism augments the efficacy of oxaliplatin-based chemotherapy in gastrointestinal cancers. Cancer Lett (2020) 473:74–89. doi: 10.1016/j.canlet.2019.12.036 31904482

[49] Jensen-Jarolim E Bax HJ Bianchini R Capron M Corrigan C Castells M. AllergoOncology – the impact of allergy in oncology: EAACI position paper. Allergy (2017) 72(6):866–87. doi: 10.1111/all.13119 PMC5498751 28032353

[50] Guan X Liu Y Xin W Qin S Gong S Xiao T. Activation of EP4 alleviates AKI-to-CKD transition through inducing CPT2-mediated lipophagy in renal macrophages. Front Pharmacol (2022) 13:1030800. doi: 10.3389/fphar.2022.1030800 36467025 PMC9709464

[51] Lopez-Castejon G Brough D. Understanding the mechanism of IL-1β secretion. Cytokine Growth Factor Rev (2011) 22(4):189–95. doi: 10.1016/j.cytogfr.2011.10.001 PMC3714593 22019906

[52] Collins SL Oh MH Sun IH Chan-Li Y Zhao L Powell JD. mTORC1 signaling regulates proinflammatory macrophage function and metabolism. J Immunol (2021) 207(3):913–22. doi: 10.4049/jimmunol.2100230 34290107

[53] Zhang Y Huang H Coleman M Ziemys A Gopal P Kazmi SM. VEGFR2 activity on myeloid cells mediates immune suppression in the tumor microenvironment. JCI Insight (2021) 6(23). doi: 10.1172/jci.insight.150735 PMC8675197 34673569

[54] Lai Y Wahyuningtyas R Aui S Chang K. Autocrine VEGF signalling on M2 macrophages regulates PD-L1 expression for immunomodulation of T cells. J Cell Mol Med (2019) 23(2):1257–67. doi: 10.1111/jcmm.14027 PMC6349155 30456891

[55] Nishikoba N Kumagai K Kanmura S Nakamura Y Ono M Eguchi H. HGF-MET signaling shifts M1 macrophages toward an M2-like phenotype through PI3K-mediated induction of arginase-1 expression. Front Immunol (2020) 11:2135. doi: 10.3389/fimmu.2020.02135 32983173 PMC7492554

[56] Coudriet GM He J Trucco M Mars WM Piganelli JD. Hepatocyte growth factor modulates interleukin-6 production in bone marrow derived macrophages: Implications for inflammatory mediated diseases. PloS One (2010) 5(11):e15384. doi: 10.1371/journal.pone.0015384 21072211 PMC2970559

[57] Finney AC Funk SD Green JM Yurdagul A Rana MA Pistorius R. EphA2 expression regulates inflammation and fibroproliferative remodeling in atherosclerosis. Circulation (2017) 136(6):566–82. doi: 10.1161/CIRCULATIONAHA.116.026644 PMC5548618 28487392

[58] Bros M Haas K Moll L Grabbe S. RhoA as a key regulator of innate and adaptive immunity. Cells (2019) 8(7):733. doi: 10.3390/cells8070733 31319592 PMC6678964

[59] Ridley AJ. Rho proteins, PI 3-kinases, and monocyte/macrophage motility. FEBS Letters. (2001) 498(2):168–71. doi: 10.1016/S0014-5793(01)02481-4 11412850

[60] Zhang J Zhu J Bu X Cushion M Kinane TB Avraham H. Cdc42 and RhoB activation are required for mannose receptor-mediated phagocytosis by human alveolar macrophages. MBoC (2005) 16(2):824–34. doi: 10.1091/mbc.e04-06-0463 PMC545914 15574879

[61] Chiou SJ Ko HJ Hwang CC Hong YR. The double-edged sword of Beta2-microglobulin in antibacterial properties and amyloid fibril-mediated cytotoxicity. Int J Mol Sci (2021) 22(12):6330. doi: 10.3390/ijms22126330 34199259 PMC8231965

[62] van Leent MMT Beldman TJ Toner YC Lameijer MA Rother N Bekkering S. Prosaposin mediates inflammation in atherosclerosis. Sci Transl Med (2021) 13(584):eabe1433. doi: 10.1126/scitranslmed.abe1433 33692130 PMC8209679

[63] Deschamps D DeBeco V Fisch C Fromenty B Guillouzo A Pessayre D. Inhibition by perhexiline of oxidative phosphorylation and the beta-oxidation of fatty acids: possible role in pseudoalcoholic liver lesions. Hepatology (1994) 19(4):948–61. doi: 10.1002/hep.1840190422 8138270

