# Supplementary material for: Proteomic characterisation of perhexiline treatment on THP-1 M1 macrophage differentiation
Source: Front Immunol. 2023 Mar 13;14:1054588. doi: 10.3389/fimmu.2023.1054588 (PMC10040681; doi:10.3389/fimmu.2023.1054588)
Supplement: Supplementary file 1 [file DataSheet_1.docx]

Supplementary Material

# Supplementary Figures


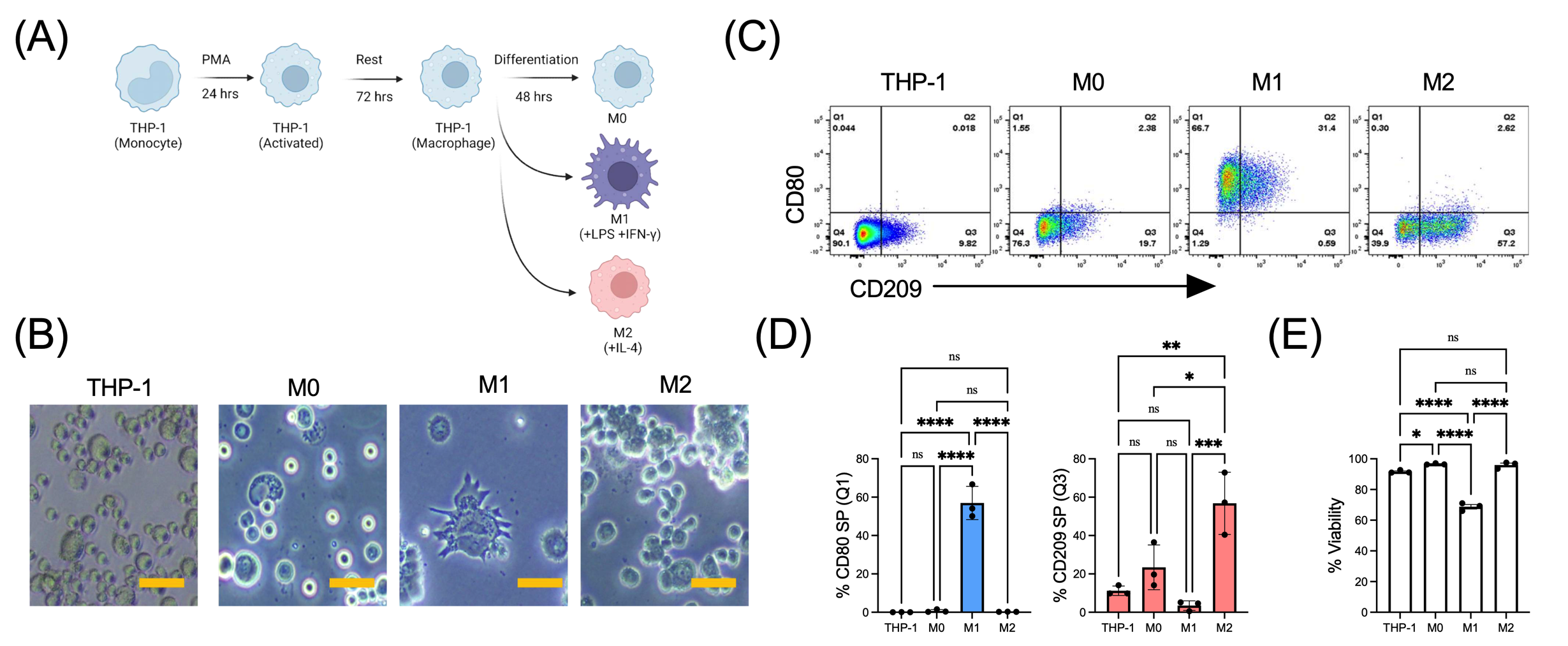


**Supplementary Figure 1. THP-1 monocyte differentiation into macrophage subsets.** (**A**) Schematic diagram for generating M0, M1 and M2 macrophages. THP-1 monocytes were stimulated with PMA for 24 hours, rested for 72 hours and incubated with media (M0), media supplemented with 250 ng/mL of LPS and 20 ng/mL of IFNγ (M1), or with 20 ng/mL of IL-4 (M2) for 48 hours. (**B**) Representative images showing the morphology of THP-1, M0, M1 and M2 macrophages. Yellow bar represents 50 μm. (**C**) Flow cytometry plots showing the expression of CD80 and CD209 for THP-1 and differentiated macrophages. (**D**) Quantitation of % CD80 single positive (SP, Q1 gate) and % CD209 SP (Q3 gate). (**E**) Viability of cultured cells. Each point represents one independent experiment and shown as mean ± SD of replicates pooled from three independent experiments. One-way ANOVA with Dunnett's multiple comparisons test was performed for the analysis **p* ≤ 0.05; ***p* ≤ 0.01; ****p* ≤ 0.001; *****p* ≤ 0.0001.


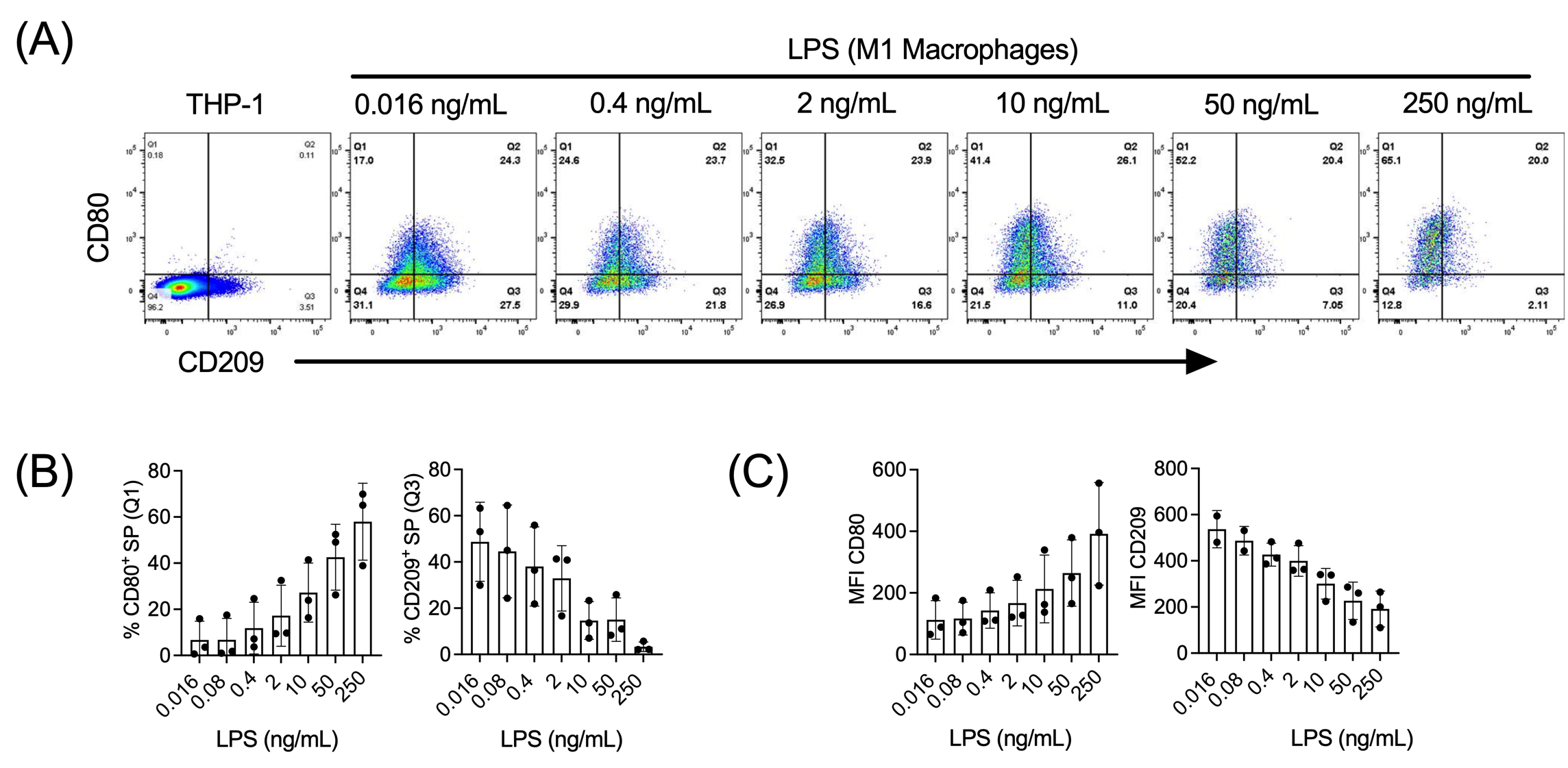


**Supplementary Figure 2. Higher concentration of LPS favors M1 macrophage differentiation.** THP-1 monocytes were stimulated with PMA for 24 hours, rested for 72 hours and incubated with various concentrations of LPS and expression of surface marker for M1 and M2 macrophages were analyzed using a flow cytometer (A) Flow cytometry plot showing the expression CD80 and CD209 (B) Quantification of % CD80 single positive (SP, Q1 gate) and % CD209 SP (Q3 gate) with varying concentration of LPS. (C) MFI values of CD80 and CD209 of M1 macrophages. Data is representative of three independent experiments bar plots show individual values and mean± SD of three independent experiments.


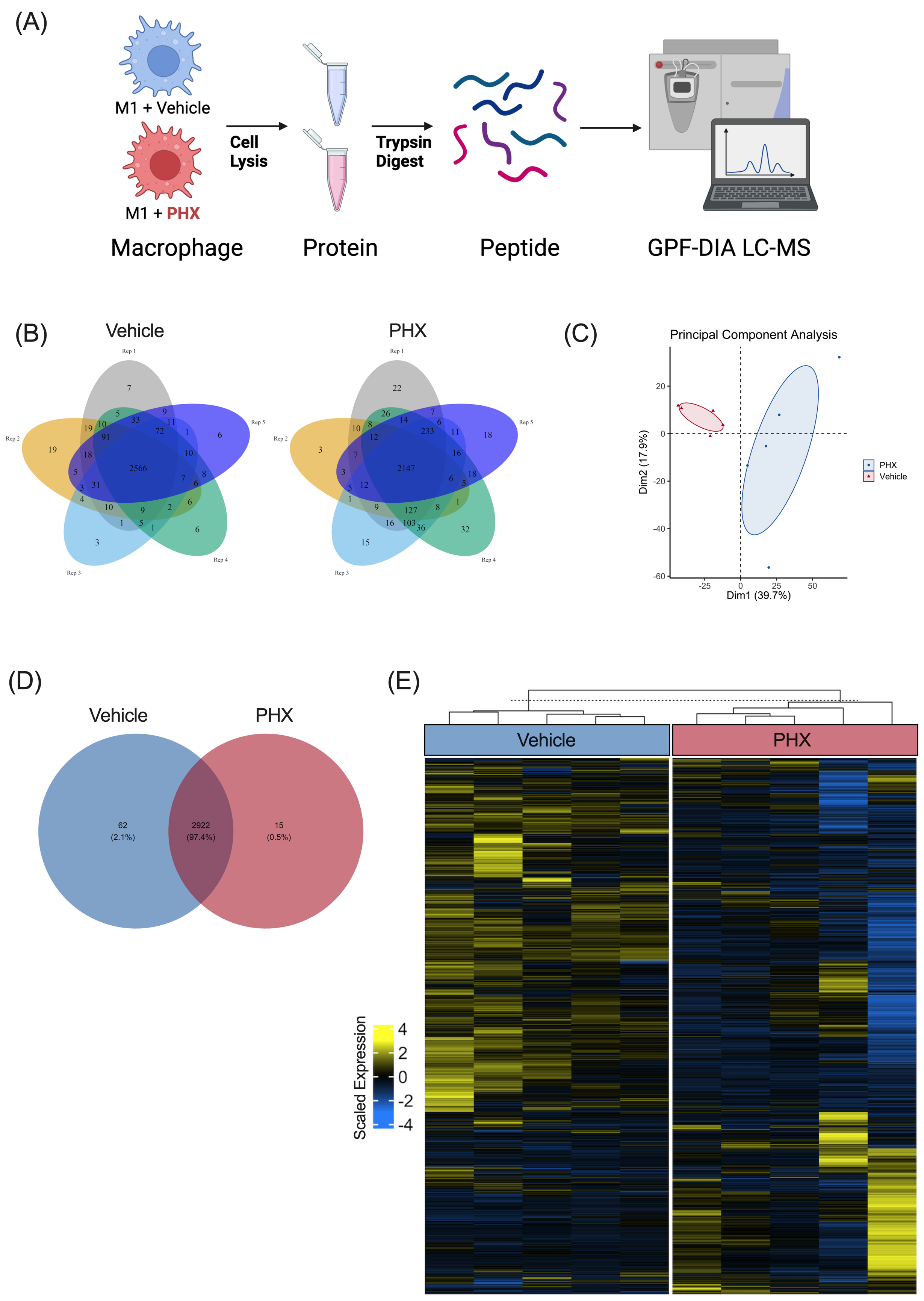


**Supplementary Figure 3. Workflow and validation of GPF-DIA proteomics on PHX treated M1 macrophages.** THP-1 derived M1 macrophages were treated with either vehicle or 5 μM PHX during differentiation and proteomic analyses of cell lysates was performed. (A) Schematic diagram of the workflow for this study. (B) Venn diagrams showing shared proteins detected among 5 replicates between vehicle and PHX treated M1 macrophages. (C) Principal Component Analysis (PCA) plot of proteomic data set comprised of 5 replicates between vehicle (control) groups and PHX (treatment) groups. (D) Venn diagram showing shared and unique proteins observed between Vehicle and PHX treated M1 macrophages. (E) Heatmap showing protein expression among 5 replicates between vehicle and PHX treated M1 macrophages.


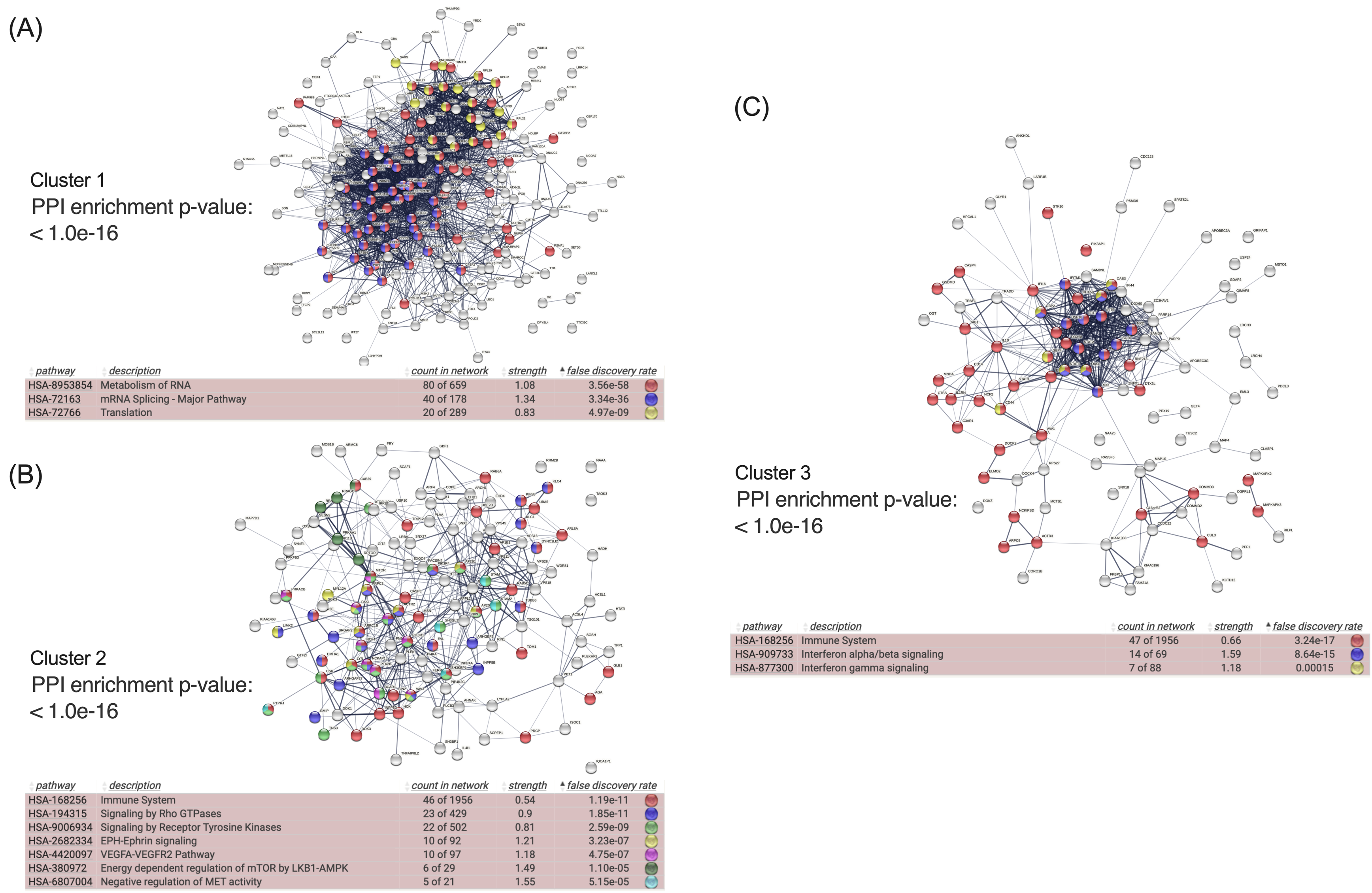


**Supplementary Figure 4. Protein-protein interaction (PPI) network analysis of significantly downregulated protein clusters in PHX treated M1 Macrophages.** Significantly downregulated proteins based on DEqMS analysis was subjected to PPI network analysis using STRING database. 3 clusters (A-C) identified were then re-analysed using PPI network analysis, with Reactome Knowledgebase used to identify significantly enriched gene sets found in each cluster.


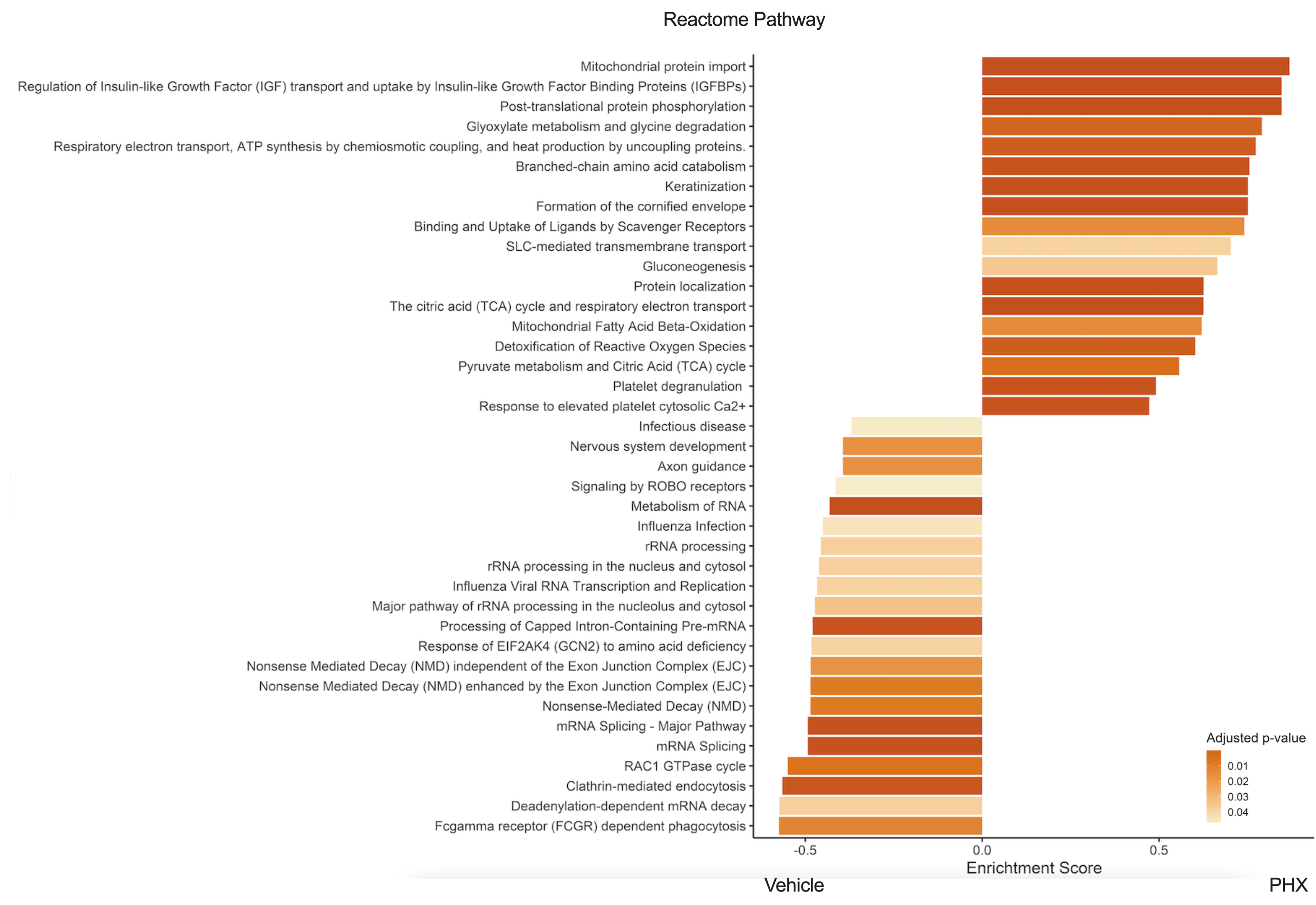


**Supplementary Figure 5. Reactome Pathway gene set enrichment analysis (GSEA) of differentially expressed proteins between vehicle and PHX treated M1 macrophages.**


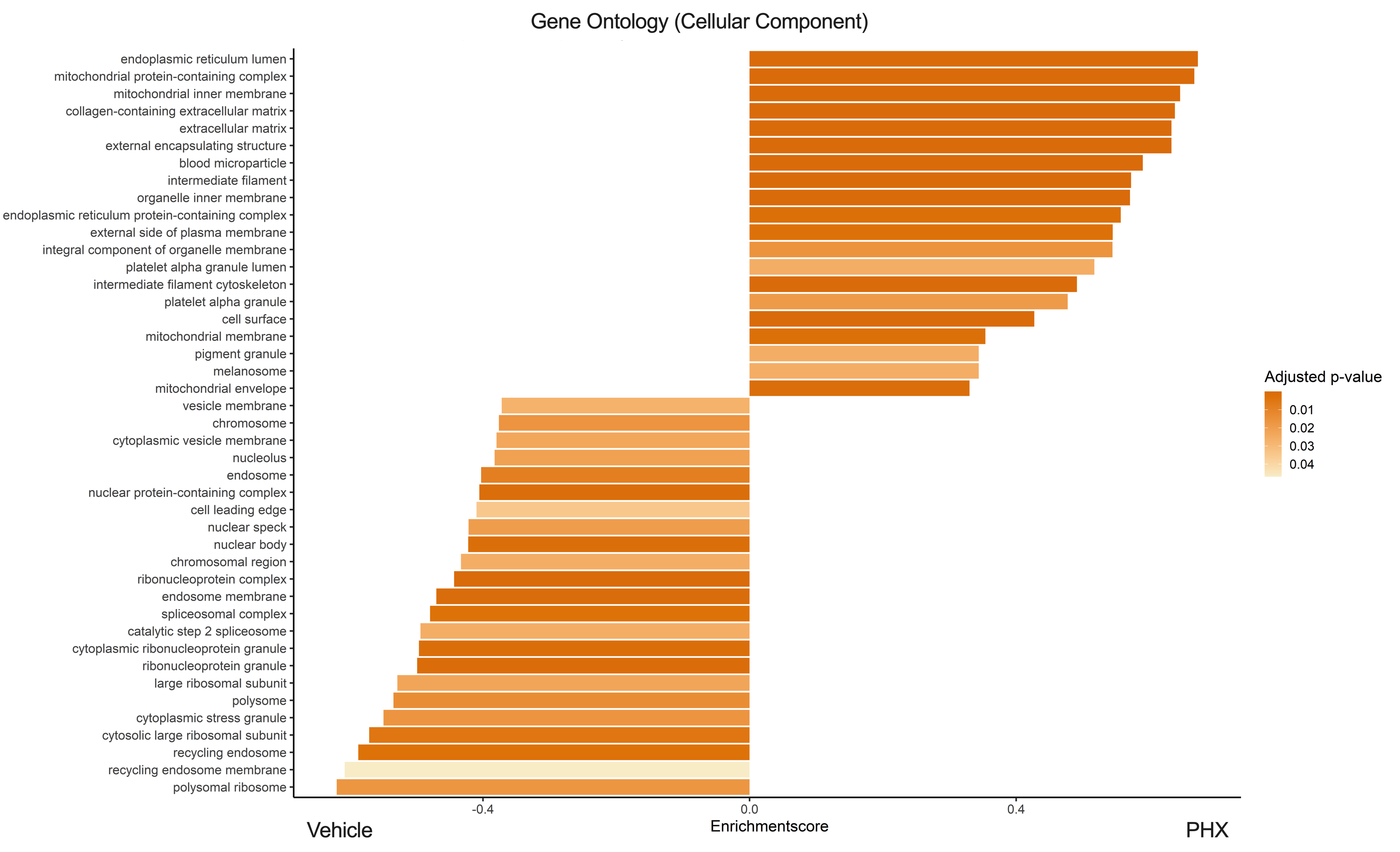


**Supplementary Figure 6. Gene Ontology (Cellular Component) GSEA of differentially expressed proteins between vehicle and PHX treated M1 macrophages.**


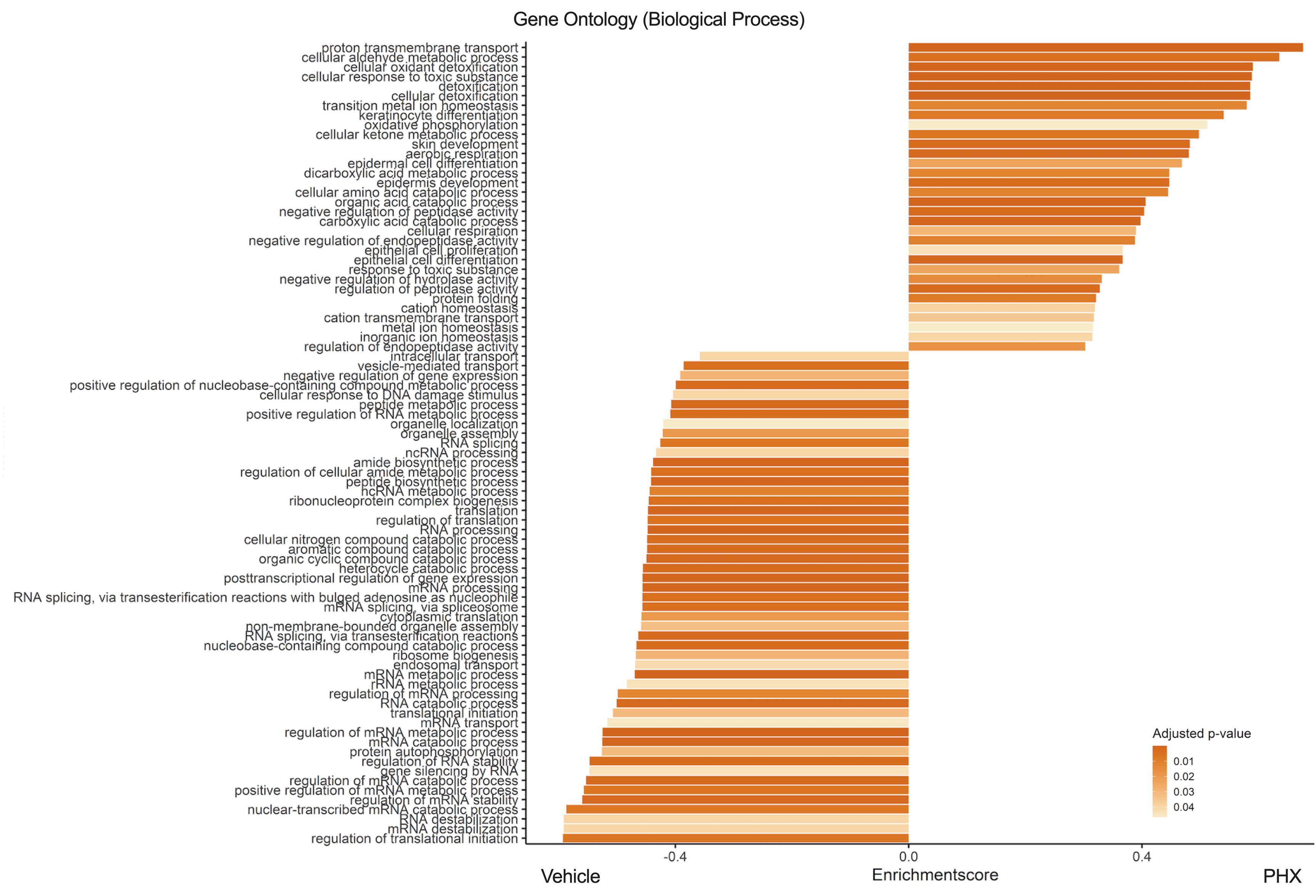


**Supplementary Figure 7. Gene Ontology (Biological Process) GSEA of differentially expressed proteins between vehicle and PHX treated M1 macrophages.**


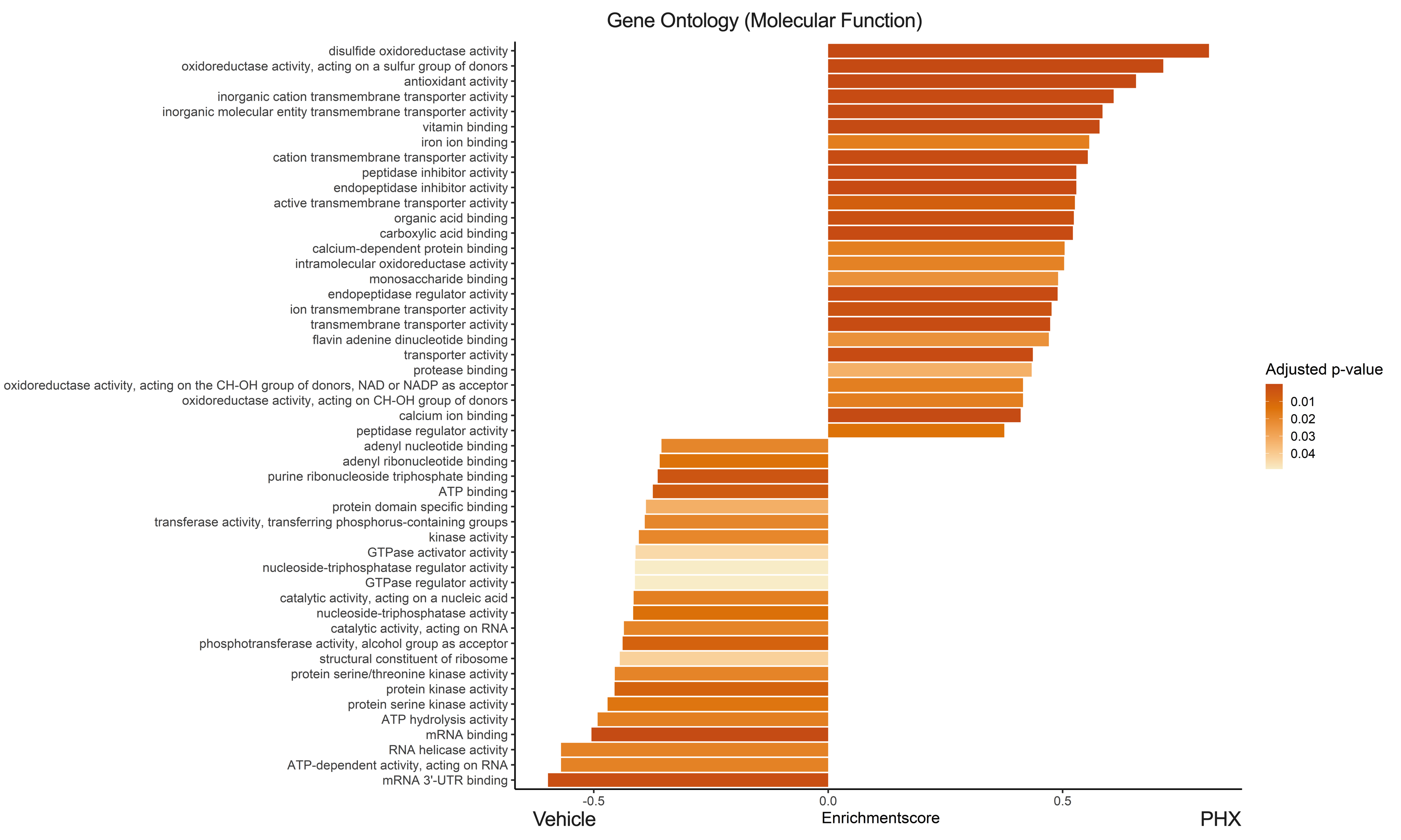


**Supplementary Figure 8. Gene Ontology (Molecular Function) GSEA of differentially expressed proteins between vehicle and PHX treated M1 macrophages.**
